# Antibacterial mechanism of hops β-acids against methicillin-resistant *Staphylococcus aureus* and promote wound healing

**DOI:** 10.3389/fmicb.2025.1710545

**Published:** 2025-12-03

**Authors:** Shuanghe Li, Shijie Wei, Feiyan Zhang, Qi Luo, Nan Yang, Xiao Zhang, Jiayue Liu, Xia Qiao, Bingren Tian

**Affiliations:** 1 Ningxia Key Laboratory of Clinical and Pathogenic Microbiology, Surgery of Laboratory, Institute of Medical Sciences, General Hospital of Ningxia Medical University, Yinchuan, Ningxia, China; 2 Department of Pharmacy, General Hospital of Ningxia Medical University, Yinchuan, Ningxia, China; 3 Sanitation Test Center, Pingliang Center for Disease Control and Prevention, Pingliang, Gansu, China; 4 The State Key Laboratory of Mechanism and Quality of Chinese Medicine, Institute of Chinese Medical Sciences, University of Macau, Taipa, Macao SAR, China

**Keywords:** β-acids, antibacterial activity, MRSA, antibacterial mechanism, wound healing

## Abstract

**Introduction:**

Skin wounds infected by Methicillin-resistant staphylococcus aureus (MRSA) still remain an important clinical challenge. β-acids, secondary metabolites extracted from hops, exhibit a powerful antibacterial effect on MRSA.

**Methods:**

In this study, the antimicrobial activity of β-acids against MRSA and the corresponding mechanism were studied.

**Results:**

The antimicrobial activity results revealed that β-acids was sensitive to MRSA (MIC = 62.5 μg/mL and MBC = 250 μg/mL). After β-acids treatment, the morphological changes of MRSA, including surface roughness, cellular crumpling, and fracture. The content of extracellular macromolecules, such as nucleic acids and proteins, increased significantly when β-acids treatment was applied, indicating that the integrity and permeability of cell membranes were disrupted. Meanwhile, laser confocal microscopy results showed that the cell membrane was severely damaged. Additionally, β-acids minimized intracellular adenosine triphosphate (ATP), suppressed Na^+^-K^+^-ATPase and Ca^2+^-Mg^2+^-ATPase activities, and accelerated intracellular reactive oxygen species (ROS) accumulation, ultimately inducing bacterial death. The result of transcriptome RNA sequencing suggested that the pathways involved “carbohydrate metabolism”, “amino acid metabolism”, “energy metabolism”, and “translation”, were significantly enriched. After the mice were successfully modeled with MRSA-infected wounds, β-acids was administered for 15 days, and faster healing of the wound area was observed in the treated group. H&E staining demonstrated gradual regeneration of dermal and epidermal tissues after β-acids treatment. Immunofluorescence results demonstrated that β-acids accelerated wound healing by markedly reducing IL-6 and TNF-α, increasing VEGF levels, and suppressing infection.

**Discussion:**

Overall, β-acids may be promising agents for the effective treatment of MRSA.

## 1 Introduction

*Staphylococcus aureus* (*S. aureus*) remains a formidable human pathogen, responsible for a spectrum of infections ranging from superficial skin lesions to life-threatening sepsis, pneumonia, and endocarditis (Lakhundi and Zhang, 2018). The emergence of methicillin-resistant *S. aureus* (MRSA), often exhibited multidrug resistance, has intensified the global health crisis (Peacock and Paterson, 2015). MRSA infections, particularly in healthcare settings (HA-MRSA) and communities (CA-MRSA), are associated with high morbidity, mortality, and treatment costs (David and Daum, 2010). Meanwhile, chronic wounds infected with MRSA exhibit delayed healing due to persistent inflammation, biofilm formation, and bacterial toxin-driven tissue damage (Pranantyo et al., 2024; Leaper et al., 2015; Foster, 2019). Conventional antibiotics often fail against biofilms, and rising resistance further limits therapeutic options. Despite the development of newer agents like linezolid and daptomycin, resistance evolution and the paucity of novel antimicrobial classes underscore the urgent need for alternative therapeutic strategies (Kawasuji et al., 2023). MRSA resistance, cytotoxicity to host cells, and disruption of beneficial microbiota (Turner et al., 2019). Biofilm-penetrating agents remain scarce, while anti-inflammatory or pro-healing effects are rarely combined with antimicrobial action. Therefore, developing alternatives to existing antibiotics for MRSA infection remains a concern.

A large number of modern studies have shown that many natural products with antibacterial activity are used as alternative drugs to fight drug-resistant pathogens (Gao et al., 2024; Lin et al., 2018; Smith et al., 2025). Therefore, investigating the relevant antibacterial mechanisms of various natural products is conducive to the development of new antibacterial drugs. However, the majority of existing studies primarily concentrate on bacterial cell membrane damage, inhibition of gene expression, and resistance to biofilm formation (Roy et al., 2018; Lu et al., 2019). Current research on carbohydrate metabolism remains limited. As a core metabolic function in bacteria, carbohydrate metabolism provides essential energy and produces metabolites that promote bacterial proliferation (Ahmad et al., 2025; Deutscher et al., 2006). Many researches have demonstrated that disruptions in carbohydrate metabolism may represent a potential inhibitory mechanism, diminishing bacterial resistance and resulting in bacterial lysis (Ritter and Wong, 2001; Peng et al., 2015). Therefore, studying carbohydrate abnormalities in bacterial metabolism by different natural active products has gradually become a research hotspot. Among them, the phosphoenolpyruvate-mediated phosphate transferase (PTS) system unique to the microbial structure is its sugar uptake system, which is responsible for the uptake of sugars and the regulation of carbohydrate metabolism (Xu et al., 2023; Assoni et al., 2020). Within the phosphotransferase system (PTS), the mannose transporter (ManP) exhibits broad substrate specificity, accommodating glucose, mannose, fructose, and glucosamine (Liu et al., 2019). Liu et al. (2019) selected cryoelectron microscopy to determine the three-dimensional structure of ManP in *E. coli*, in which the study pointed out ManP could transport substrates through cell membranes through an “elevator” mechanism. Sun et al. (2023) found that magnolol blocks MRSA’s nutrient supply by inhibiting carbohydrate metabolism, causing bacterial lysis and disintegration. Thus, ManP might serve as a potential target for magnolol in combating MRSA infections.

Hops are widely grown perennial crops, where the soft resins in them are usually discarded as industrial waste after harvest (Rossini et al., 2021). It was reported that there is a large number of active ingredients—β-acids in soft resins. β-acids are secondary metabolites extracted from hops, which include lupulone, colupulone, and adlupulone (Zhang et al., 2021). Numerous studies have demonstrated that β-acids exhibit significant antibacterial activity on both Gram-positive and Gram-negative bacteria (Tian et al., 2021; Tian et al., 2024; Goedseels and Michiels, 2023; Liu et al., 2025). In our previous research, β-acids were found to disrupt cell membrane integrity, leading to cell death (Tian et al., 2022). Additionally, the hydrophobic structure of β-acids is capable of disrupting the cell membrane of *S. aureus* (Kolenc et al., 2022). While these findings indicate that β-acids have a potent inhibitory effect on *S. aureus*, further investigation is required to elucidate the inhibitory mechanisms of β-acids against MRSA, particularly concerning their impact on biofilm formation and host-pathogen interactions. It is worth noting that although new evidence suggests that some pyramid compounds have anti-inflammatory properties, the comprehensive potential of β-acids to inhibit MRSA and accelerate infected wound healing has not been explored (Sommella et al., 2021). In a word, while β-acids’ baseline antibacterial effects are acknowledged. However, there is no study on the antibacterial mechanism of β-acids on MRSA that involves ManP to inhibit carbohydrate metabolism.

This study investigated the antibacterial activity and mechanism of β-acids against MRSA. Through determining the minimum inhibitory concentration (MIC) and growth curve, the antibacterial efficacy was evaluated. Its mechanism was explored by examining the effects of β-acids on MRSA’s cell morphology, membrane permeability, membrane integrity, and bacterial metabolism. Additionally, the therapeutic effect of β-acids in MRSA-infected mouse wound models was further assessed by monitoring wound healing and changes in inflammatory factors. By integrating the antibacterial mechanisms with wound healing outcomes, this study provides a basis for developing β-acids as a multifunctional therapeutic agent for MRSA-infected wounds. It also contributes to promoting the application of β-acids as a potential antibacterial agent against MRSA.

## 2 Experiment

### 2.1 Materials and reagents

Hops extract (obtained via supercritical CO₂ extraction) was supplied by Xinjiang Sapporo Agricultural Science and Technology Development Co., Ltd. (Xinjiang, China), while β-acids were extracted in our laboratory (Tian et al., 2021). Sodium hydroxide and acetic acid (99.5% purity) were purchased from Damao Chemical Reagent Co., Ltd. (Tianjin, China). Agar powder (1,300 g/cm^2^ purity) was acquired from Coolaber Science & Technology Co., Ltd. (Beijing, China). Tryptone and yeast extract were sourced from Oxoid Ltd. (United Kingdom). Alkaline phosphatase (AKP) and ATPase activity assay kits were obtained from Abbkine Scientific Co., Ltd. The reactive oxygen species kit, LIVE/DEAD Bacterial Staining Kit (with DMAO & PI), and ATP assay kit were provided by Beyotime Biotechnology (Nanjing, China). The bicinchoninic acid (BCA) protein assay kit was supplied by KeyGEN Biotechnology Co., Ltd. (Nanjing, China), and 1% Crystal Violet Ammonium Oxalate Solution was purchased from Beijing Solarbio Science & Technology Co., Ltd. (Beijing, China). The MRSA standard strain (ATCC33591) was obtained from Jiangxi Zhonghong Boyuan Biotechnology Co., Ltd. Pentobarbital sodium was supported by Sigma Co., Ltd.

### 2.2 LC-MS analysis

Raw extracts were analyzed by LC-MS using a Waters ACQUITY^™^ UPLC system coupled with a SYNAPT^™^ G2-Si Q-TOF high-definition mass spectrometer (Waters Corp., Manchester, United Kingdom). β-acids separation was achieved on an ACQUITY UPLC HSS T3 column (2.1 × 100 mm, 1.8 μm) at 30 °C. The mobile phase consisted of 0.1% (v/v) aqueous formic acid (A) and acetonitrile (B), with a gradient elution program at a flow rate of 0.3 mL/min: 30–64% B (0–2 min); 64–68% B (2–10 min); 68–70% B (10–40 min); 70–90% B (40–42 min).

The mass spectrometer was configured to operate in positive and negative ionization modes, with data acquisition conducted in centroid mode across a mass-to-charge (*m*/*z*) range of 100 to 1,500. The instrument parameters were established as follows: a capillary voltage of 3 kV for positive electrospray ionization (ESI^+^) and 1.5 kV for negative electrospray ionization (ESI^−^); a cone voltage of 40 V; an ion source temperature of 120 °C; a desolvation temperature of 350 °C; a cone gas flow rate of 40 L/h; and a desolvation gas flow rate of 450 L/h.

### 2.3 Determination of MIC and MBC of β-acids

The minimum inhibitory concentration (MIC) of β-acids against MRSA was analyzed by the broth dilution method. Briefly, β-acids were dissolved in liquid medium containing 1% DMSO and 1% Tween 80, then serially diluted in a 96-well plate to obtain concentrations ranging from 4 mg/mL to 156.25 μg/mL. Subsequently, 100 μL of MRSA suspension (1 × 10^6^ CFU/mL) was put into every well, and the plate was put at 37 °C for 24 h. The lowest β-acids concentration showing no visible bacterial growth to the naked eye was regarded as MIC. For determining MBC, 100 μL aliquots from each well were plated on Luria-Bertani (LB) agar and incubated at 37 °C for 24 h; the lowest concentration with no visible bacterial growth was considered as MBC.

### 2.4 Determination of time-kill curve

β-acids were added into 5.0 mL MRSA suspensions (1 × 10^6^ CFU/mL) so as to prepare treatment groups with concentrations corresponding to 1/4, 1/2, 1, and 2MIC, followed by incubation at 37 °C. The control group contained no β-acids. At 0 h, 2 h, 4 h, 6 h, 8 h, 12 h, the bacterial growth was recorded via determining its absorbance at 600 nm. Three replicates were set for each group.

### 2.5 Scanning electron microscopy analysis

Morphological changes in MRSA were examined by scanning electron microscopy (SEM). Briefly, MRSA without β-acids treatment served as the control, while those treated with β-acids at the MIC (62.5 μg/mL) formed the experimental group; both were incubated at 37 °C. After 12 h, bacterial cells were fixed in 2.5% glutaraldehyde at 4 °C overnight, then rinsed three times with PBS and dehydrated using a graded ethanol series (25, 50, 75, 90, 100%), with each step lasting 10 min. After pre-freezed at −80 °C, samples were freeze-dried for 12 h, finally subjected to vacuum sputter gold coating, and observed via SEM.

### 2.6 Cell wall damage assays

Alkaline phosphatase (AKP) leakage assay was performed to assess the impact of β-acids on the cell wall of MRSA (Yi et al., 2024). Specifically, β-acids were added to 5.0 mL MRSA suspensions (1 × 10^6^ CFU/mL) so as to prepare treatment groups with concentrations of 1/4, 1/2, 1, and 2MIC, whereas the control group contained no β-acids. All samples were incubated at 37 °C, and the supernatant was obtained every 2 h for 8 h to measure AKP activity with a commercial detection kit. Each experiment was repeated three times.

### 2.7 Biofilm inhibition assay

The antibiofilm efficacy of β-acids was assessed utilizing the crystal violet staining method. MRSA was inoculated into 96-well plates and incubated for 24 h. Then, Luria-Bertani (LB) medium containing β-acids at varying concentrations (1/16, 1/8, 1/4, and 1/2 of the MIC) was introduced to the bacterial cultures, followed by an 18 h incubation period at 37 °C. The control group was devoid of β-acids. Following incubation, the culture medium was removed, and the bacterial cells were washed with phosphate-buffered saline before being stained with a 0.1% crystal violet solution for 30 min. After staining, the cells underwent three washes with sterile PBS and were subsequently decolorized with 95% ethanol for 30 min. The optical density was then measured at 595 nm. The absorbance percentage relative to the control group was calculated based on these measurements.

### 2.8 Membrane integrity analysis

Bacterial membrane integrity was evaluated using dual-fluorescence staining with 4′,6-diamidino-2-phenylindole (DAPI) and propidium iodide (PI) as previously described with modifications (Kong et al., 2024). Briefly, MRSA cells (OD = 0.6) were collected and washed with PBS. Then bacteria were treated with β-acids at sub-inhibitory to bactericidal concentrations (1/2MIC, 1MIC, 2MIC) for 2 h at 37 °C. Following incubation, bacteria were washed and co-stained with DAPI (5 μg/mL) and PI (30 μg/mL) in PBS for 15 min at 25 °C in the dark. Cells were then washed twice to remove excess dyes, resuspended in PBS, and mounted on glass slides. Fluorescence images were captured with Leica SP8 confocal laser scanning microscope with excitation/emission settings of 358/461 nm (DAPI; blue) and 535/617 nm (PI; red).

### 2.9 Bacterial biofilm formation and harvesting

The inhibitory effect of β-acids on the formation of MRSA biofilms was quantified utilizing the crystal violet (CV) staining method, as previously outlined (O’Toole, 2011). In summary, MRSA cultures with an optical density (OD) of 0.6 were diluted at a ratio of 1:100 in fresh tryptic soy broth supplemented with 1% glucose. Aliquots of 200 μL were distributed into sterile 96-well polystyrene plates and treated with sub-inhibitory concentrations of β-acids, specifically at 1/16MIC, 1/8MIC, 1/4MIC, 1/2MIC, 1MIC, and 2MIC, to evaluate biofilm suppression without eliciting bactericidal effects. After 24 h of static incubation at 37 °C, planktonic cells were removed, and the adherent biofilms were gently washed twice with phosphate-buffered saline. The biofilms were subsequently fixed with 200 μL of methanol for 15 min, stained with a 0.1% (w/v) CV solution for 30 min, and thoroughly rinsed with deionized water. The bound CV was solubilized in 200 μL of 30% (v/v) glacial acetic acid, and the absorbance was measured at 595 nm using a microplate reader.

### 2.10 Determination of the ATP level

β-acids were introduced into the bacterial culture medium at final concentrations corresponding to 0.5, 1, 2, and 4 times MIC. Subsequently, the cultures were incubated for 2 h at 37 °C with agitation at 180 revolutions per minute. Following incubation, the cultures were subjected to centrifugation at 5,000 × g for 5 min to pellet the bacterial cells, which were subsequently washed with sterile phosphate-buffered saline. The supernatant was collected for extracellular ATP level determination using an ATP detection kit. Concurrently, the bacterial pellet was lysed with lysozyme, and the resulting supernatant was collected to measure intracellular ATP levels.

### 2.11 Determination of ATPase activity

MRSA was exposed to varying concentrations of β-acids (0, 1/2MIC, 1MIC, and 2MIC) for a duration of 2 h at a constant agitation speed of 150 revolutions per minute, maintained at a temperature of 37 °C. Bacteria were collected by centrifugation at 8,000 rpm for 4 min, washed twice, and resuspended in 0.85% NaCl solution. Bacterial cells were disrupted using a probe sonar in an ice water bath. After that, the samples were centrifuged at 10,000 rpm for 10 min (4 °C), and the supernatant was extracted. Protein concentration in the supernatant was determined by BCA assay. The activities of Na^+^-K^+^-ATPase and Ca^2+^-Mg^2+^-ATPase were measured using the ATPase activity assay kit.

### 2.12 Reactive oxygen species detection

The influence of β-acids on intracellular reactive oxygen species (ROS) levels in MRSA was assessed utilizing the DCFH-DA fluorescent probe. MRSA cultures were subjected to various concentrations of β-acids (0, 1/2MIC, 1MIC, and 2MIC) for a duration of 2 h at 37 °C, with agitation at 150 rpm. Subsequently, the bacterial cells were harvested via centrifugation at 5,000 g for 5 min, washed with sterile phosphate-buffered saline, and resuspended in 1 mL of phosphate buffer containing 10 μM DCFH-DA. The suspension was then incubated for 30 min at 37 °C in the dark, with continuous shaking at 150 rpm. Following centrifugation to pellet the cells, the intracellular ROS levels were quantified by measuring the fluorescence intensity of the samples at a wavelength of 525 nm.

### 2.13 Determination of nucleic acid and proteins release

Bacterial suspensions were cultured to the logarithmic growth phase in LB medium, subsequently harvested, and subjected to three washes with 10 μM phosphate-buffered saline (pH 7.4). The bacteria were then resuspended in a 0.9% sodium chloride solution and exposed to varying concentrations of β-acids, specifically 0, 1/2MIC, 1MIC, and 2MIC. At specified intervals (0, 2, 4, 6, and 8 h), samples were collected through centrifugation at 8,000 revolutions per minute for 10 min. The absorbance of the resulting supernatants was measured at wavelengths of 260 nm and 280 nm.

### 2.14 Protein content determination by SDS-PAGE

The MRSA (1 × 10^8^–10^9^ CFU/mL) was inoculated into LB medium and cultured at 37 °C for 16 h. Then, different concentrations of β-acids (1/4MIC, 1/2MIC, 1MIC and 2MIC) were added to the LB medium containing MRSA, and PBS was taken as a control. After 2 h, collect the cell precipitate by centrifuging at 4,000 rpm for 10 min, and suspend it in 100 μL of PBS and the protein loading buffer was added. The mixture was heated in boiling water for 8 min, then centrifuge at 8,000 rpm for 10 min. Finally, 20 μL of the supernatant from each sample was added for SDS-PAGE analysis. After electrophoresis, stain with Coomassie Blue R-250 for 20 min, and separate the protein bands using a defoamer.

### 2.15 Transcriptome analysis

Total RNA was isolated from both the treatment (T-group) and control (C-group) groups utilizing a commercial RNA extraction kit, following the manufacturer’s instructions. The purity and concentration of the extracted RNA were determined using a Nanodrop spectrophotometer (Thermo Fisher Scientific, Wilmington, United States), while RNA integrity was evaluated with an Agilent 2100 Bioanalyzer (Agilent Technologies, United States). RNA sequencing libraries for both groups, comprising three biological replicates per group, were subsequently constructed on the Illumina HiSeq^™^ 2500 platform (Novogene, China). To ensure high-quality data, raw sequencing reads were processed by removing adaptor sequences, reads containing more than 10% unknown bases (N), and reads with over 50% low-quality bases (*Q*-value ≤10). For gene expression analysis, unigene expression levels were quantified using the FPKM (fragments per kilobase of transcript per million mapped reads) metric. Differentially expressed genes (DEGs) between the samples were identified using DESeq2 software, with significant DEGs defined by an absolute fold change of ≥2 and an adjusted *p*-value of <0.05. Functional categorization of these DEGs was performed using Gene Ontology (GO) and Kyoto Encyclopedia of Genes and Genomes (KEGG) analyses.

### 2.16 Molecular docking and dynamics simulations

The crystal structure of the protein exhibiting the highest sequence homology to ManP (PDB code: 6K1H) was selected from the RCSB Protein Data Bank. The structural model of ManP from MRSA was subsequently constructed using the Swiss-Model platform (available at https://swissmodel.expasy.org/). The structures of β-acids were generated using ChemDraw and subsequently optimized. Molecular docking studies were conducted using Discovery Studio 2020 (BIOVIA, CA, United States). The optimal binding conformation of β-acids to ManP was determined through binding energy calculations. Further analysis and visualization were performed using PyMOL version 2.0.6 (Schrödinger, MA, United States).

### 2.17 *In vivo* wound healing analysis

Animals (SPF-grade C57BL/6J mice) were housed in a facility at 23 ± 2 °C and 50 ± 5% relative humidity. All the animals were provided with sufficient distilled water and standard rodent feed. Following a one-week period of adaptive feeding, the mice were randomly allocated into two groups: the Control group (*n* = 3) and the β-acids group (*n* = 3). Post-anesthesia (pentobarbital sodium, 20 mg/kg, injected), the dorsal hair of the mice was shaved, and a wound with a 10-millimeter diameter was created using surgical scissors. Each wound was inoculated with 100 μL of an MRSA suspension at a concentration of 1 × 10^6^ CFU. Subsequent to infection, 20 μL of PBS (0.9%) and β-acids (12.5 μg/mL) were applied to the wounds, respectively. On days 3, 6, 9, 12, and 15, wound size was assessed by photographing the treated areas. After 15 days, the mice were euthanized (pentobarbital sodium, 60 mg/kg, injected).

Tissue samples were fixed in 10% neutral buffered formalin, embedded in paraffin at 25 °C, and subjected to standard hematoxylin and eosin (H&E) staining. To evaluate growth factor expression in skin tissues and assess the wound-healing efficacy of the treatments, the tissues were stained according to the established protocol and examined microscopically.

### 2.18 Statistical data analysis

All experiments were done in triplicates, and data were expressed as means ± SD (*n* = 3). Visualized using GraphPad Prism 9.0 and Origin 8.6.0. Statistical significance was analyzed via one-way ANOVA, with significance defined as *p* < 0.05.

## 3 Results and discussion

### 3.1 Chemical components identification

The compositional analysis conducted via UPLC-Q-TOF/MS revealed that hops β-acids are predominantly composed of three compounds: colupulone (molecular weight: 400.26), lupulone (molecular weight: 414.27), and adlupulone (molecular weight: 414.27) (Supplementary Figure S1), based on MS fragment and reference compounds comparison. The purity of the hops β-acids was quantified at 97.41% by the LC-MS linear normalization method. There are three peaks prominent in the total ion chromatography of β-acids, with retention times of 18.13, 22.63, and 23.88 min, respectively. The above results are consistent with Gao’s et al. (2024) analysis of the active ingredients in hops.

### 3.2 Antibacterial activity of β-acids against MRSA

**Figure 1:**
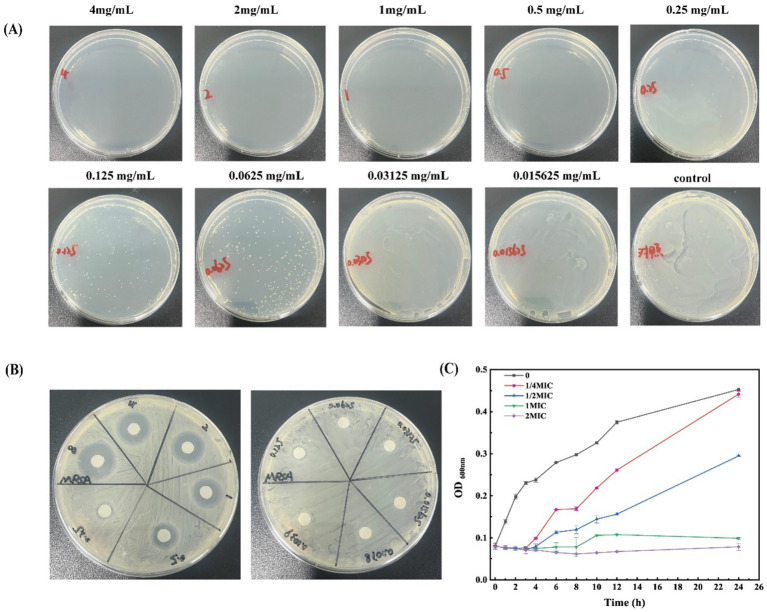
Antibacterial activity of β-acids against MRSA. **(A)** MBC of β-acids. **(B)** Inhibition zone of β-acids. **(C)** The growth curve of MRSA under different dosing of β-acids.

The MIC of β-acids against MRSA, determined via broth microdilution, was 62.5 μg/mL, while the MBC measured by the same method was 250 μg/mL (Figure 1A). Additionally, the filter paper method was used to verify the MIC, yielding a consistent result of 62.5 μg/mL (Figure 1B). Relevant studies have shown that Magnolia officinalis, oridonin, lactobionic acid, and *Litsea cubeba* essential oil at varying concentrations all exert significant inhibitory effects on MRSA growth, with their MICs being 20 μg/mL, 64 μg/mL, 18.75 mg/mL, and 0.5 mg/mL, and MBCs being 40 μg/mL, 512 μg/mL, 50 mg/mL, and 1.0 mg/mL, respectively (Sun et al., 2023; Yuan et al., 2019; Kang et al., 2020; Hu et al., 2019). A comparison with these reported data revealed that β-acids displayed stronger inhibitory activity against MRSA than lactobionic acid and *Litsea cubeba* essential oil.

Furthermore, growth curves of MRSA were determined at different β-acids concentrations to investigate the antibacterial activity (Figure 1C). It can be seen that the bacteria showed a rapid growth trend within 12 h without β-acids. However, as the concentration of β-acids increased, the growth of the bacteria was inhibited. In the concentration of 1/4MIC, bacteria started multiplying at 3 h. After 24 h, the inhibition rate was 18.14%. Compared with the untreated samples, the growth rate was decreased, indicating that β-acids inhibited the proliferation of MRSA. When the β-acids concentration increased to 1MIC, the growth of MRSA was inhibited significantly (the inhibition rate was 87.79%). Only a slight increase in the bacterial population was observed at 8 h. Nevertheless, when the addition concentration of β-acids was 2MIC (125 μg/mL), the inhibition rate of 24 h was 97.56%, indicating that the bacterial proliferation was almost completely inhibited. It was found that β-acids could effectively inhibit the growth of MRSA with concentration-dependent. Higher concentrations of β-acids suppressed the growth of MRSA more effectively.

### 3.3 Antibacterial mechanism of β-acids against MRSA

#### 3.3.1 Effect of β-acids on morphology of MRSA

**Figure 2:**
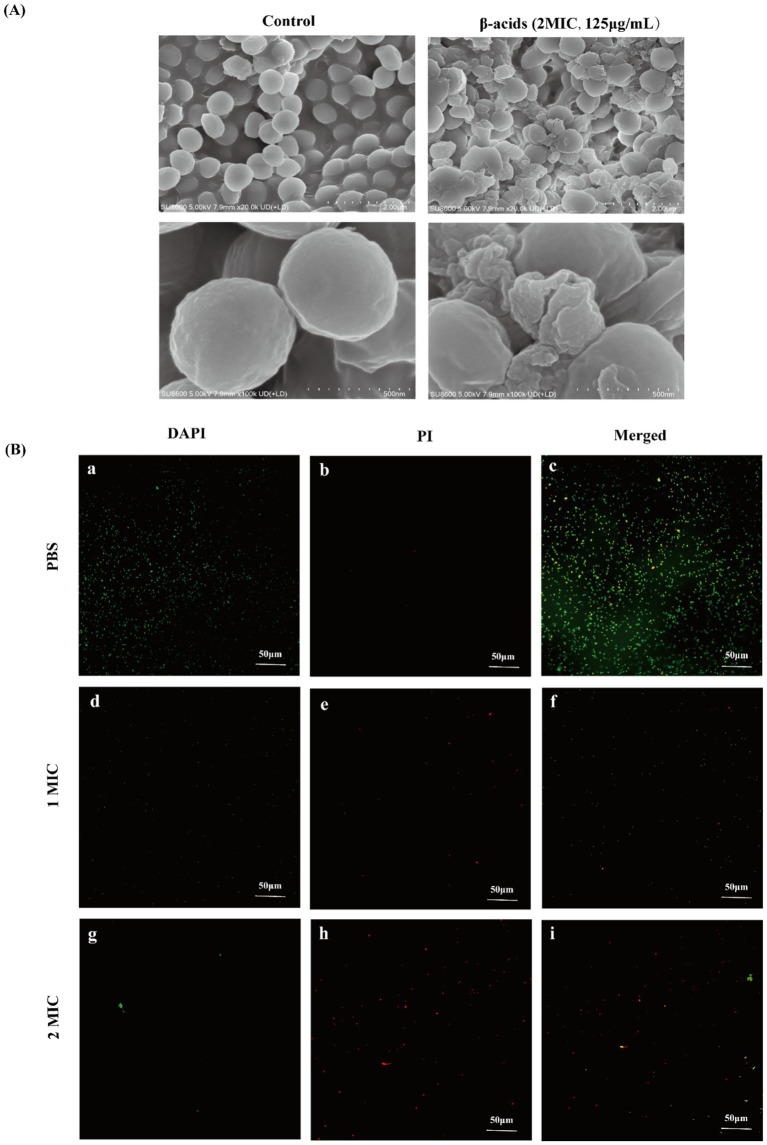
SEM and laser confocal microscopy images of MRSA. **(A)** SEM of MRSA. **(B)** Laser confocal microscopy images of MRSA after treatment with β-acids at different concentrations for 4 h.

When bacteria were inhibited or killed by antibacterial agents, the morphology usually changed, such as contraction, deformation, lysis of the cytoplasm, formation of pores on the membrane, and cell disintegration (Kong et al., 2024; Weng et al., 2024). As shown in Figure 2A, the morphological changes of MRSA after treatment with β-acids were observed by SEM. The untreated cells were in the form of regular spheres, with a round shape, smooth surface and intact overall structure. On the contrary, after being treated with β-acids at 2MIC, the overall structure of the bacteria was disrupted. It was observed that the cell membranes ruptured, holes formed on the membranes, severe deformations and adhesion in adjacent cells be noticed. In addition, the cell surface and its surroundings showed no visible particles before treatment. However, after treatment with β-acids, many irregular particles were found on the surface, which may originate from the protoplasm that leaks out when bacterial cells rupture. The results indicated that β-acids could cause the leakage of intracellular substances by disrupting the integrity of the cell surface structure, thereby killing the cells. Similar studies also prove that after having the active ingredient effect, the appearance and shape of MRSA cells have undergone varying degrees. For example, after Tao et al. (2021) treated with MIC concentration of total flavonoid extract of white ermatitis, MRSA cells were uneven in size, and vacuole-like irregular circular structures appeared in large quantities, with rough cell surfaces, loose cell wall structures, and overflow of cell contents.

#### 3.3.2 Effect of β-acids on cell membrane integrity

The integrity of the cell membrane of MRSA after being treated with β-acids was studied by using the laser confocal microscope. DAPI and PI were used as nucleic-acid dyes to differentiate between intact and damaged bacterial cell membranes (Tao et al., 2025). PI could only penetrate the damaged cell membrane and trigger red fluorescence, which can be used as an indicator of “dead” cells. DAPI could permeate into bacteria and generate a green fluorescence. As shown in Figure 2, fluorescence microscopy images were collected after treated with β-acids at varying concentrations, displayed that the bacteria treated with PBS all emitted green fluorescence (Figures 2B,a), with the red fluorescence being the least (Figures 2B,b). It could be found that the cell membranes of the bacteria were all intact, with little PI entering the cell. In contrast to the control group (PBS), the green fluorescence of the cells treated with 1MIC reduced (Figures 2B,d) and the red fluorescence was significantly enhanced (Figures 2B,e), indicating that the cell membrane integrity of almost half of the bacteria was disrupted. When treating the bacteria with 2MIC, the vast majority of the cells emitted red fluorescence (Figures 2B,h). This demonstrated that high concentrations of β-acids could lead to severe disruption of cell membrane integrity, which was consistent with the results of SEM and extracellular protein content and nucleic acid levels. β-acids disrupted MRSA in a concentration-dependent manner.

#### 3.3.3 Effect of β-acids on MRSA biofilm formation

**Figure 3:**
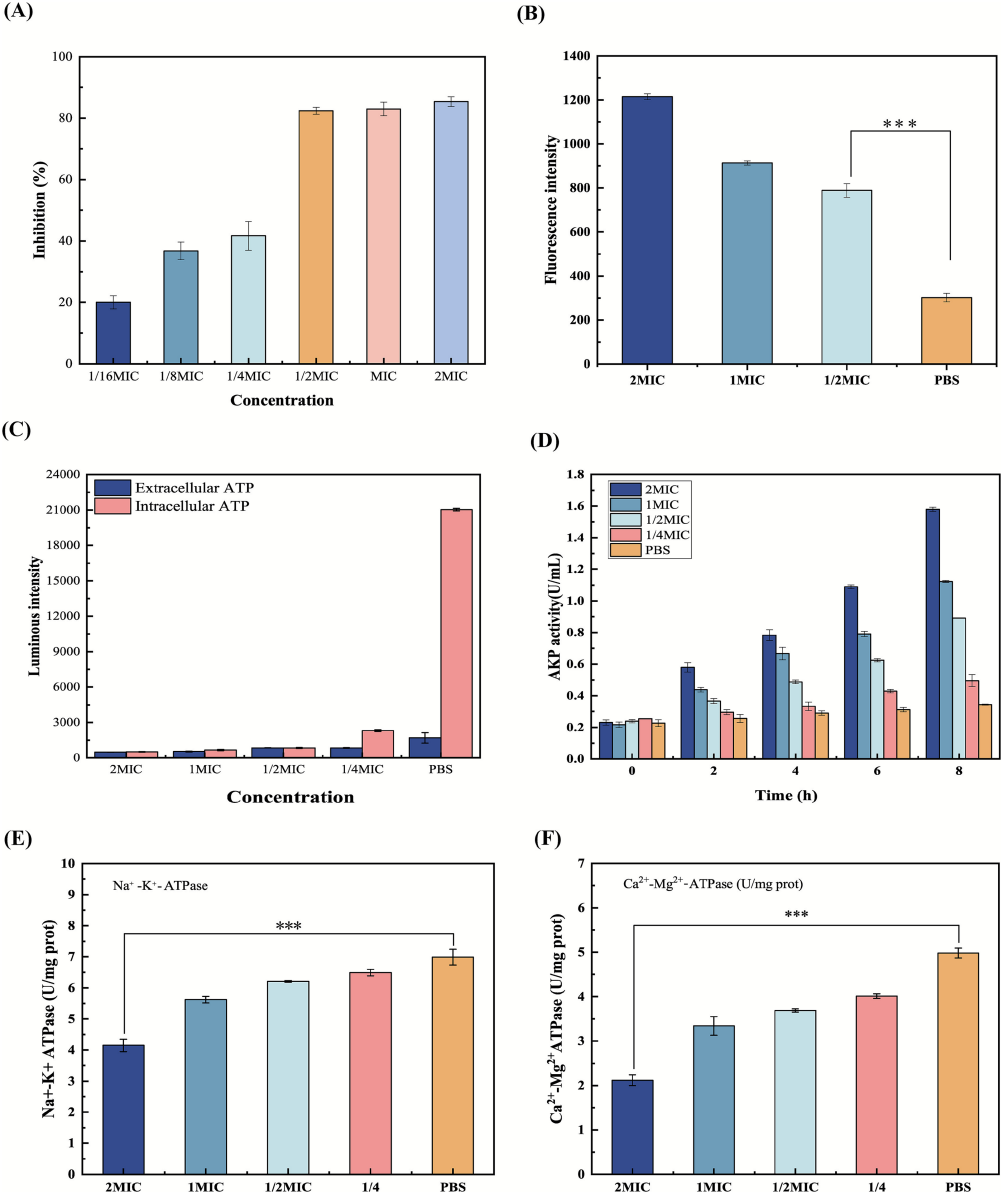
**(A)** Inhibition of β-acids on MRSA biofilm formation. **(B)** The intracellular ROS level. **(C)** ATP level of MRSA. **(D)** AKP activity of MRSA. **(E)** Na^+^-K^+^-ATPase of MRSA. **(F)** Ca^2+^-Mg^2+^-ATPase of MRSA. ^***^p ≤ 0.001.

Biofilms create a protective microenvironment that supports bacterial survival, explaining why most pathogenic and environmental bacteria reside in biofilm communities (Baeza and Mercade, 2021). Biofilm formation is a dynamic, continuous regulatory process, primarily determined by its structure, function, and composition (Baeza and Mercade, 2021). The crystal violet staining means was used to evaluate the impact of β-acids on forming MRSA biofilm, with results presented in Figure 3A. Treatment of MRSA with β-acids at 1/16MIC inhibited biofilm formation by 20.0%, while at 1/2MIC, the inhibition rate significantly increased to 82.4%. However, at concentrations of 1MIC and 2MIC, the inhibition rate showed little change. This suggests that β-acids at 1/2MIC can inhibit biofilm formation in most bacteria, consistent with findings from cell membrane integrity studies. Thus, β-acids exert their antimicrobial effects partly by suppressing MRSA biofilm formation.

#### 3.3.4 Effects of β-acids on the intracellular ROS level

ROS, a highly reactive oxygen-derived molecule that is the secondary metabolite in the process of aerobic metabolism, which often associated with cellular damage and oxidative stress (Averill-Bates, 2024). In normal cells, ROS is maintained at a low level to act as a signaling molecule (Soheili et al., 2022). However, when cells are damaged, the balance between ROS production and antioxidant defense is disrupted, with excessive accumulation of intracellular ROS. In this study, the levels of ROS in bacterial cells were determined after treatment with different concentrations of β-acids, shown in Figure 3B.

In the absence of β-acids treatment, ROS was maintained at a low level. In contrast, when treated with β-acids, intracellular ROS levels increased with the concentration of β-acids. Oxidative stress occurred when ROS levels increased and exceeded the capacity of the cellular antioxidant defense system, causing damage to macromolecules, such as proteins, DNA, and lipids (Tikhomirova et al., 2024). This demonstrated that β-acids would promote intracellular ROS accumulation and cause cellular damage.

#### 3.3.5 Effect of β-acids on the ATP level and ATPase activity

ATP is not only a universal energy carrier within the cell, but also plays an important role as an extracellular signaling molecule (Spari and Beldi, 2020). At the same time, ATP can reveal the metabolic state of bacteria. The level of intracellular ATP serves as an indicator of cell viability, which is considered to decrease when its level decreases (Kukurugya et al., 2024). Consequently, the intracellular and extracellular ATP content of bacteria treated with β-acids was determined and the results were displayed in Figure 3C. By comparison with the control group (PBS), the ATP levels of bacteria were reduced after 2 h of β-acids treatment in all cases. Specifically, the more significant the decrease in the ATP level with increasing β-acids concentration. ATP level was lowest when the β-acids concentration was 2MIC.

ATPase, a class of enzymes that catalyze the hydrolysis and synthesis of ATP and play important roles in ion transport and energy metabolism (Fedosova et al., 2022). Na^+^-K^+^-ATPase and Ca^2+^-Mg^2+^-ATPase are indispensable, serve as critical gatekeepers of bacterial ion homeostasis, energizing nutrient uptake, pH regulation, and membrane potential maintenance. Na^+^-K^+^-ATPase is located primarily in the cell membrane and maintains the gradient of Na^+^ and K^+^ across the cell membrane to facilitate ATP synthesis (Fedosova et al., 2022). Inhibiting ATPase disrupts cellular energetics, osmotic balance, and signal transduction, ultimately compromising viability (Ravera et al., 2023). Therefore, we measured the effect of different β-acids concentrations on ATPase activity after 2 h of administration, and the results were displayed in Figures 3E,F.

Na^+^-K^+^-ATPase maintains essential electrochemical gradients in bacterial cells, energizing secondary transport, osmoregulation, and membrane potential. As shown in Figure 3E, Na^+^-K^+^-ATPase activity was significantly reduced (*p* < 0.05) when administered at a concentration of 2MIC. The progressive decline of Na^+^-K^+^ ATPase’ function with increasing β-acids concentrations implies direct or indirect interference with its synthesis, stability, or catalytic activity. The results in Figure 3F suggested that Ca^2+^-Mg^2+^-ATPase activity showed the same decreasing trend. Ca^2+^ stabilizes lipopolysaccharide (LPS) in the Gram-negative outer membrane, and Mg^2+^ cross-links wall acids in the Gram-positive peptidoglycan. The decrease in Ca^2+^-Mg^2+^-ATPase activity disrupts intracellular Ca^2+^ homeostasis, leading to bacterial death. Therefore, the observed dose-dependent reduction in Ca^2+^-Mg^2+^-ATPase activity induced by the drug suggests a potent mechanism for bacterial growth inhibition. Overall, inhibition of ATPase activity may disrupt ionic homeostasis. On the one hand, intracellular ATP levels were significantly depleted, leading to impaired energy metabolism. On the other hand, the bacterial dependence on the Na^+^ gradient drives the collapse of the transporter, leading to nutrient starvation by blocking nutrient uptake and toxin efflux (Oh et al., 2011). Sun et al. (2023) investigated the mechanism of MRSA inhibition by lactobionic acid. The results similarly showed that lactobionic acid was able to reduce ATPase activity, leading to the disruption of ionic homeostasis, which in turn led to good bacterial inhibition, and proved to be a promising multifunctional food additive with antimicrobial activity.

#### 3.3.6 Effect of β-acids on cell wall damage

The cell wall is mainly composed of peptidoglycan, surface proteins and phosphate. The highly cross-linked peptidoglycan can stabilize the cell and plays an important role in maintaining the intrinsic morphology of MRSA cells (Silver, 2006). AKP, a type of enzyme, localized between the cell wall and cell membrane (Yang et al., 2022). Normally, AKP activity cannot be detected outside the cell. However, when the cell wall structure is disrupted, AKP exits the cell through the cell wall and can be detected. Consequently, the impact of β-acids on MRSA cell wall integrity can be assessed by the extracellular AKP activity assayed. As shown in Figure 3D, the bacterial AKP activity increased significantly with prolonged treatment time compared with the Control group. In particular, the increase in AKP activity was more pronounced when the administered concentration was 1MIC (*p* < 0.05). When administered at a concentration of 2MIC, AKP levels were maximized at the 8 h. Conversely, the Control group exhibited no significant alteration in AKP activity, indicating that β-acids may alter the cell wall structure of MRSA, leading to substantial leakage of AKP from the cell. This observation aligns with the findings of Kang et al. (2020). Typically, the bacterial cell wall serves as a barrier against external substances. However, upon administration of antimicrobial agents, the integrity of the bacterial cell wall is compromised. Consequently, the potent antibacterial efficacy of β-acids may be attributed to their capacity to disrupt the cell wall.

#### 3.3.7 Effect of β-acids on cell membrane permeability

**Figure 4:**
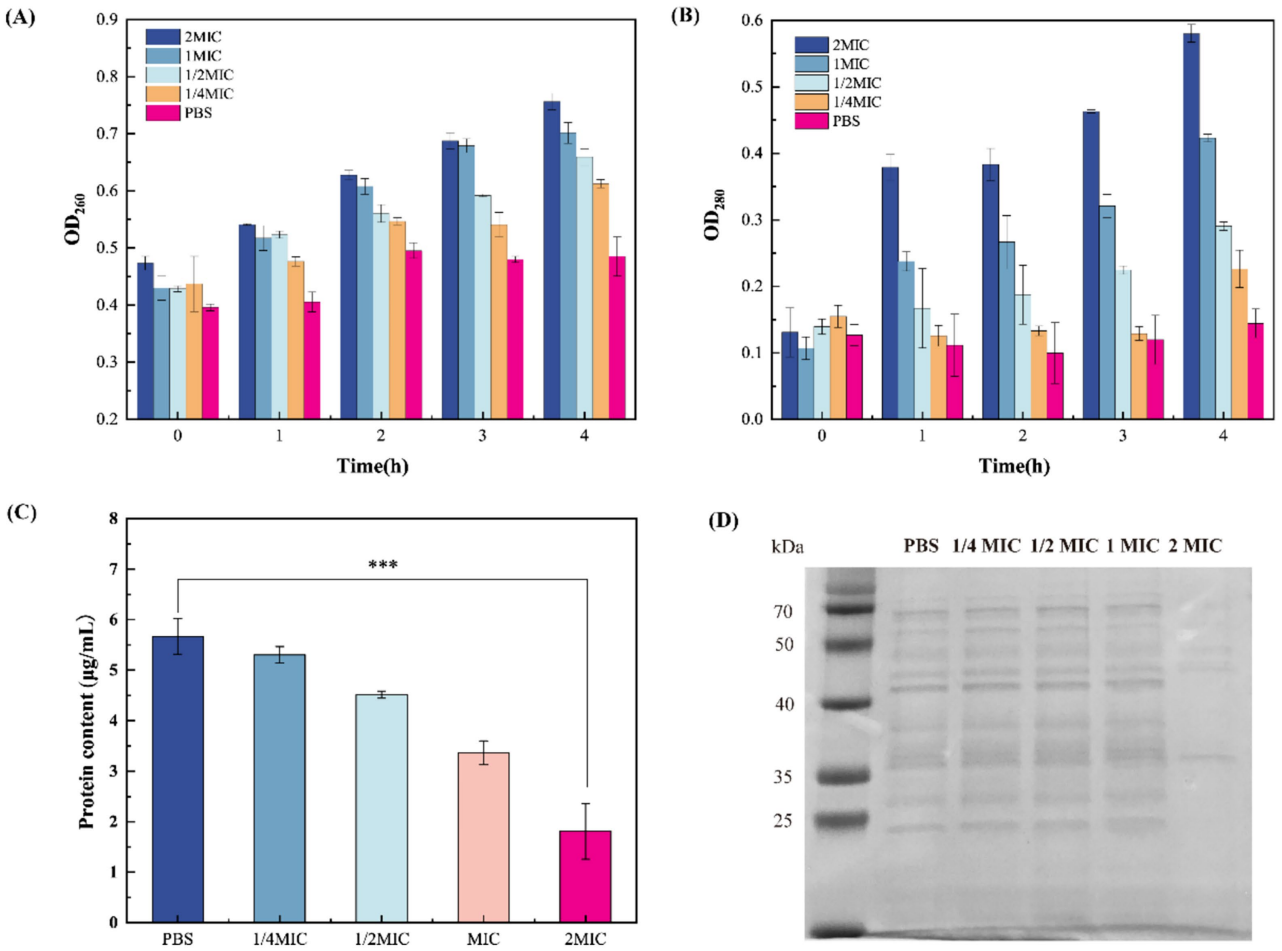
Effect of β-acids on cell membrane permeability. **(A)** The extracellular nucleic acid level of MRSA after treated with different concentration of β-acids. **(B)** The extracellular protein level of MRSA after treated with different concentration of β-acids. **(C)** Protein quantification. **(D)** SDS-PAGE profile of proteins.

The integrity of the bacterial cytoplasmic membrane is fundamental to cellular homeostasis, acting as a selective barrier that retains essential macromolecules while excluding external threats. When cell membrane permeability is compromised, the loss of permeability control leads to uncontrolled efflux of intracellular components (Broz et al., 2020). To test the hypothesis that β-acids induce damage to bacterial cell membranes, membrane permeability was assessed by quantifying the leakage of nucleic acids and protein content. Figure 4 shows the changes in extracellular nucleic acid and protein contents after treatment with different concentrations of β-acids over various incubation periods. The extracellular nucleic acid content at different times was showed in Figure 4A. It can be observed that for the bacteria treated with β-acids, the extracellular nucleic acid content increased over time. After one hour, the extracellular nucleic acids of the bacteria treated with β-acids significantly increased, while those of the untreated cells remained almost unchanged, indicating that the cell membranes were damaged. On the whole, the amount of extracellular nucleic acids in the untreated bacterial cells increased to a lesser extent than that in the treated ones. It can be concluded that β-acids could cause damage to the cell membrane, leading to the leakage of nucleic acids. Figure 4B shows the changes of the extracellular protein level as a function of time, which followed the same trend as that of extracellular nucleic acid. The results showed that after incubation for 1 h, the extracellular protein levels in the treated group significantly increased. As the concentration of β-acids increased and the time prolongs, the level of extracellular nucleic acid proteins also increased. This indicates that the cell membrane was gradually being damaged, resulting in the leakage of large molecules.

Protein is the material basis necessary for maintaining normal life activities such as physiological metabolism and energy conversion in the bacterium. In this experiment, the effect of β-acids on MRSA intracellular proteins was assessed by SDS-PAGE analyses. As shown in Figure 4D, the proteins were well separated and the bands were mainly distributed in 25–70 kDa. Compared with the control group, the color of the bands became significantly lighter or even disappeared when the concentration of β-acids was 2MIC in the treated group. It indicated that the protein content was significantly reduced, which was consistent with the results of protein quantitative detection (Figures 4C,D; Supplementary Figure S2). This may be due to the loss of intracellular proteins or the blockage of protein synthesis as a result of the breakdown of the cell membrane. A drastic reduction in protein content could disrupt the normal physiological functions of the cell, leading to rapid cell death.

### 3.4 Transcriptomic analysis of T-group and C-group

The potential antibacterial mechanism of the T-group was investigated via whole-transcriptome RNA sequencing. A total of 16.0 million and 14.1 million clean reads were generated for the T-group and C-group, respectively, with over 96.95 and 97.59% mapping to the reference genome. Expression analysis (FPKM >1) identified 2,421 expressed genes in the C-group and 1,666 in the T-group. Compared to the C-group, the T-group exhibited 1,268 differentially expressed genes (DEGs, 76.11%), comprising 609 upregulated and 659 downregulated genes.

**Figure 5:**
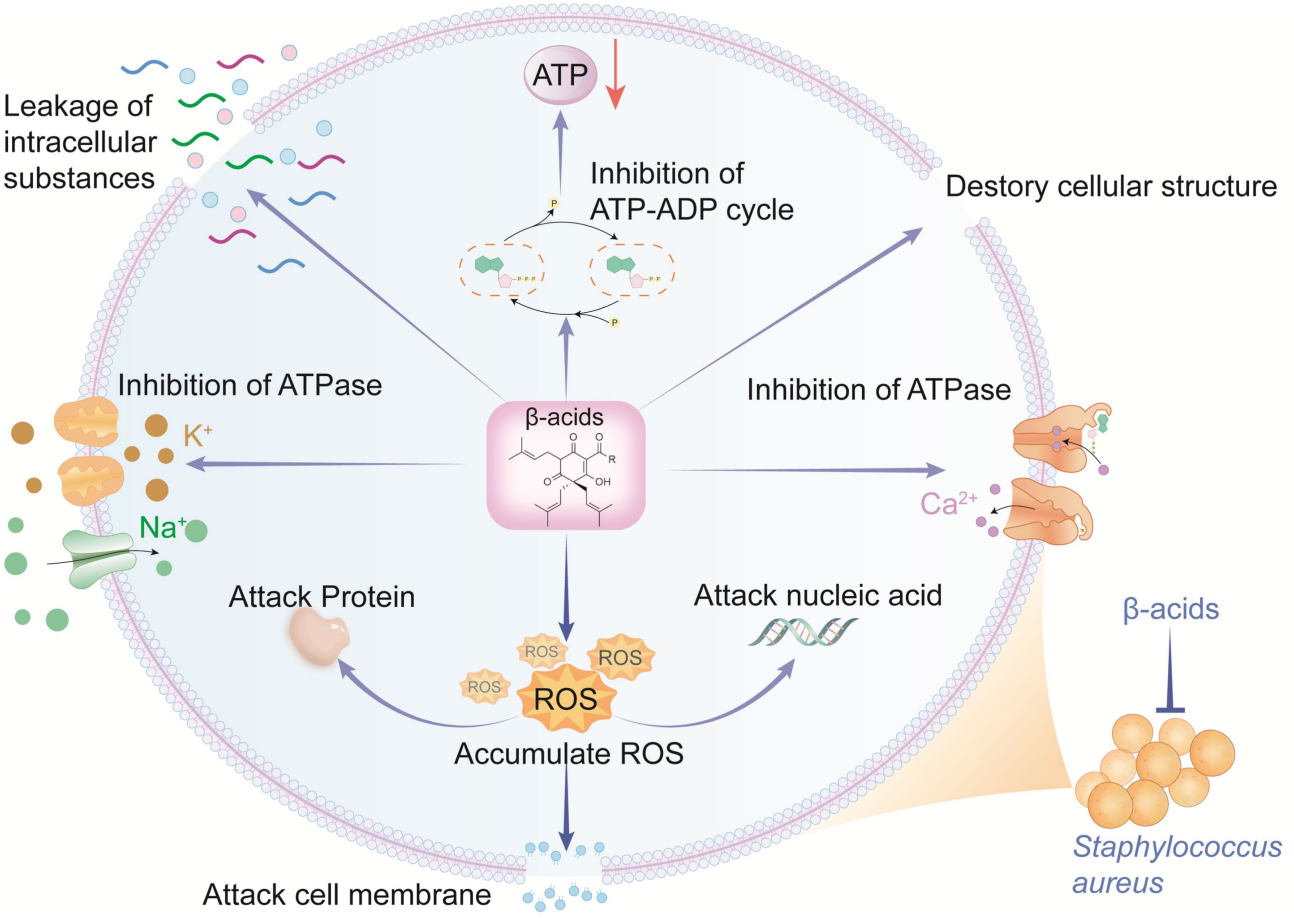
Antibacterial mode of β-acids against MRSA.

To elaborate on the functions of differentially expressed genes (DEGs), GO and KEGG enrichment analyses were performed (Supplementary Figures S3A,B). KEGG enrichment classification was applied to identify significantly enriched pathways of DEGs, revealing that all DEGs in the T-group were assigned to 70 pathways. Notably, “carbohydrate metabolism,” “amino acid metabolism,” “energy metabolism,” and “translation” were significantly enriched. Proteins with decreased expression in these key pathways (relative to the control group) are summarized in Supplementary Table S1. Energy, metabolism and oxidative stress mutually promote each other and form a lethal cycle. Specifically, β-acids affects carbohydrate metabolism, triggering “insufficient ATP synthesis” and “excessive ROS production.” Conversely, the inhibition of ATP exacerbates ROS damage, while excessive ROS production inhibits ATP synthesis (Zeng et al., 2022). Ultimately, β-acids jointly inhibits bacterial survival through energy deprivation and oxidative damage. Overall, β-acids represent a promising antibacterial candidate against MRSA, as they inhibit MRSA growth by disrupting cell membrane permeability and integrity, ion transport, energy metabolism, and reactive oxygen species balance (Figure 5). Table 1 displayed different anti-MRSA mechanisms between β-acids and other natural agents.

**Table 1:** Anti-MRSA mechanism of β-acids and other natural agents.

Anti-MRSA agents	Type of MRSA	Possible mechanism	References
*Magnolia officinalis* root extract	MRSA ATCC 43300	Inhibition of the uptake; metabolism of carbohydrates	Sun et al. (2023)
Oridonin	MRSA ATCC BAA-1717	Changes in cell membrane and cell wall permeability; disturbance in protein and DNA metabolism; influence on bacterial morphology	Yuan et al. (2019)
Lactobionic acid	MRSA N315	Disrupt the integrity of cell wall and membrane; the content and activity of bacterial proteins; bind to genomic DNA to disturb the normal cellular function	Kang et al. (2020)
*Litsea cubeba* essential oil	MRSA T29	Destructive effect on cytomembrane; intracellular enzymes leakage; inhibit the respiratory metabolism; lower the key regulator enzyme activity	Hu et al. (2019)
Aartemisinin	MRSA T29	Increase cell membrane permeability; inhibit the respiratory metabolism	Lin et al. (2018)
6-alk(en)ylsalicylic acids	MRSA ATCC 33591	Biophysical disruption of the membrane	Kubo et al. (2003)
Licochalcone A	MRSA-BH, MRSA-ZH, and USA 300	Arginine metabolism; cause the accumulation of intracellular ROS; inhibit the expression of glucokinase	Zeng et al. (2024)
β-acids	MRSA ATCC 33591	Disrupting cell membrane permeability and integrity, ion transport, energy metabolism, and reactive oxygen species balance	This study

### 3.5 Molecular docking analysis

**Figure 6:**
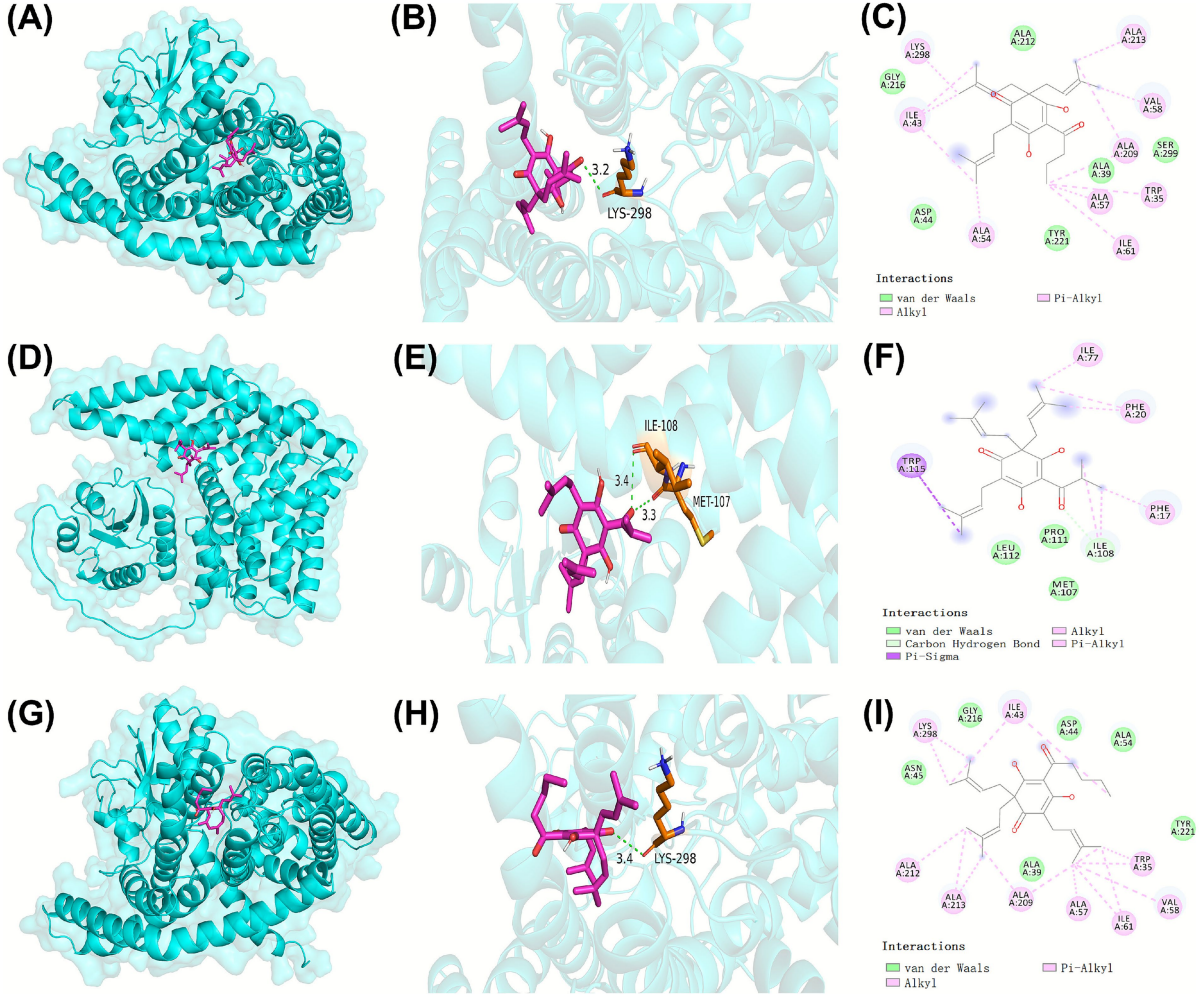
The interaction of ManP with colupulone **(A,B,C)**, lupulone **(D,E,F)**, and adlupulone **(G,H,I)**. The three-dimensional ribbon model depicted ManP, while the representative β-acids molecules including colupulone, lupulone, and adlupulone were illustrated using a rose-red stick model. The orange and blue sticks, accompanied by numerical labels, indicated the amino acid residues of ManP involved in hydrogen bonding interactions. Critical interactions between the β-acids and the active amino acid residues of ManP were highlighted using solid and dashed lines.

RNA-seq analysis demonstrated that β-acids primarily inhibited the growth of MRSA by disrupting carbohydrate metabolism. To elucidate the potential binding interactions between β-acids and ManP, molecular docking studies were conducted. Figure 6 showed that β-acids established a robust binding affinity with ManP through interactions with multiple amino acid residues. The binding energy of ManP with colupulone, lupulone, and adlupulone were −6.8, −6.5, −6.8 kcal/mol, respectively, which indicated a strong interaction between these β-acids molecules and the ManP protein.

As displayed in Figure 6, three typical molecules of β-acids, namely colupulone, lupulone, and adlupulone, exhibit strong binding affinities with ManP through interactions with multiple amino acid residues. Specifically, the oxygen atoms of colupulone and adlupulone establish conventional hydrogen bond with LYS298 similarly, while the oxygen atoms of lupulone form hydrogen bonds with ILE108 and MET107. Additionally, the side chains of colupulone, lupulone, and adlupulone engage in van der Waals, Pi-Alkyl, Alkyl, and other interactions with various amino acid residues of ManP, contributing to the stabilization of their binding with ManP. Likewise, IId mycotoxins—such as lactobactin A, lactobacillus B, and gavisin Q—have been identified to interact with amino acid residues at the active site of ManP, demonstrating broad-spectrum antibacterial activity. Given that ManP is absent in plants and animals, it presents a promising target for antibacterial drug development. β-acids have the potential to form strong interactions with ManP through hydrogen bonding, van der Waals forces, and hydrophobic interactions. Consequently, β-acids may play a significant role in the treatment of MRSA infections.

### 3.6 Effect of β-acids on MRSA-infected wound healing in animals

**Figure 7:**
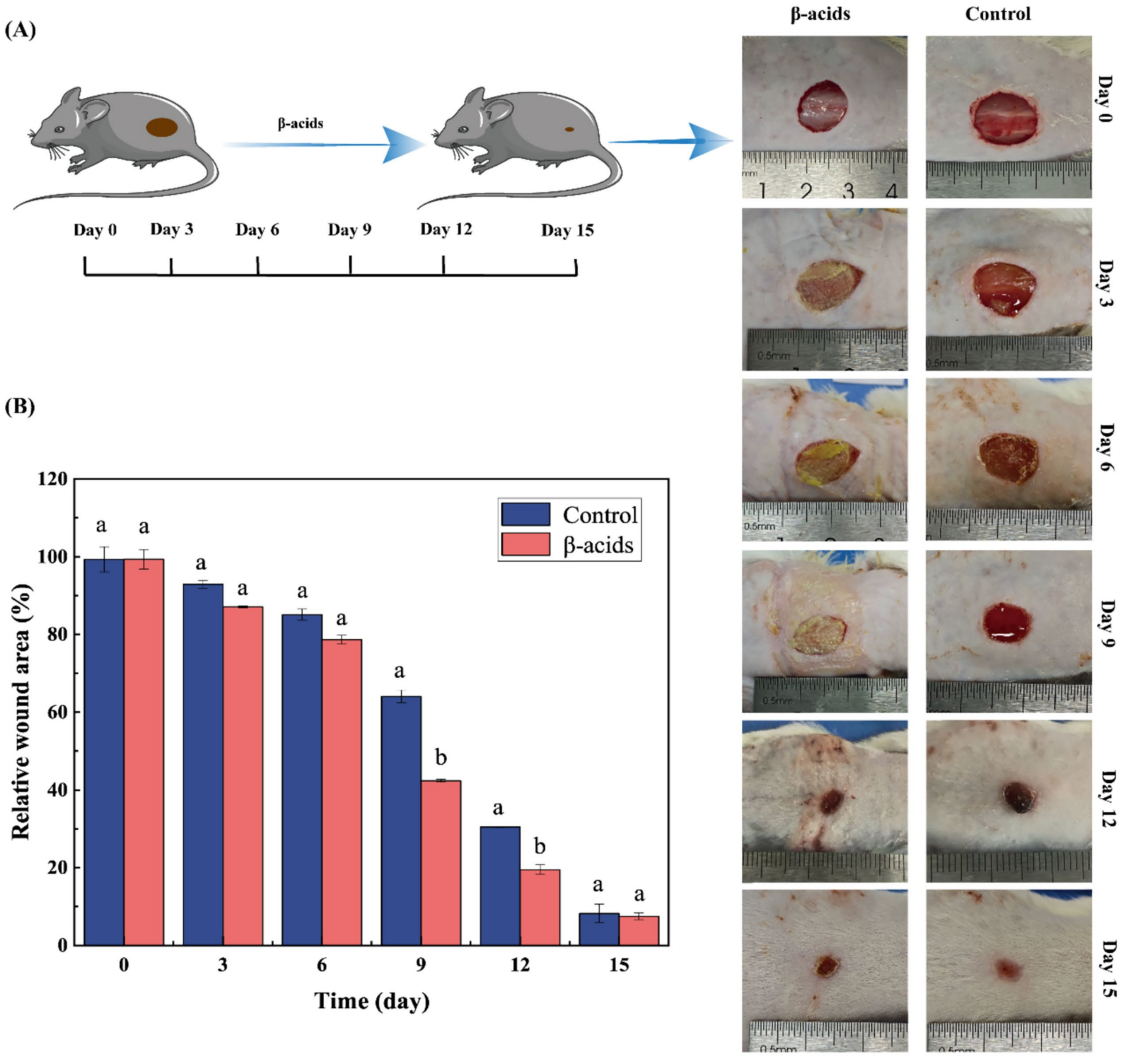
Impact of β-acids on wound healing in MRSA-infected mice. **(A)** Images of wounds on the mice’s backs. **(B)** Relative wound areas in the control and β-acids treatment groups. Data are expressed as the mean ± standard deviation from three replicate measurements. Means marked with different letters differ significantly (*p* < 0.05).

*In vivo* wound repair and antibacterial efficacy of β-acids were evaluated by MRSA-infected cutaneous wounds. Following establishment of the infected wound model, β-acids were applied to cover the wound beds, and wound healing processes were photographed over 15 days of treatment (Figure 7A). On day 3, control group wounds showed suppuration—indicating an infection-induced inflammatory response—whereas β-acids-treated wounds exhibited no yellow pus and had begun to scab gradually. Concurrently, β-acids treatment led to a gradual reduction in wound area relative to controls, reflecting effective infection control and incipient healing.

Quantitative analysis of wound area (Figure 7B) revealed initial differences in relative wound size, with a significant reduction in the treatment group by day 9, demonstrating progressive improvement in healing over time. By day 15, the relative wound area was 8.23% in controls versus 7.50% in the β-acids group, confirming that β-acids accelerate healing and promote wound contraction. Additionally, β-acids facilitated absorption of wound secretions, maintained a moist healing environment, and reduced pus exudation. These findings indicate that β-acids can effectively mitigate bacterial infection during chronic wound healing and prevent sepsis.

**Figure 8:**
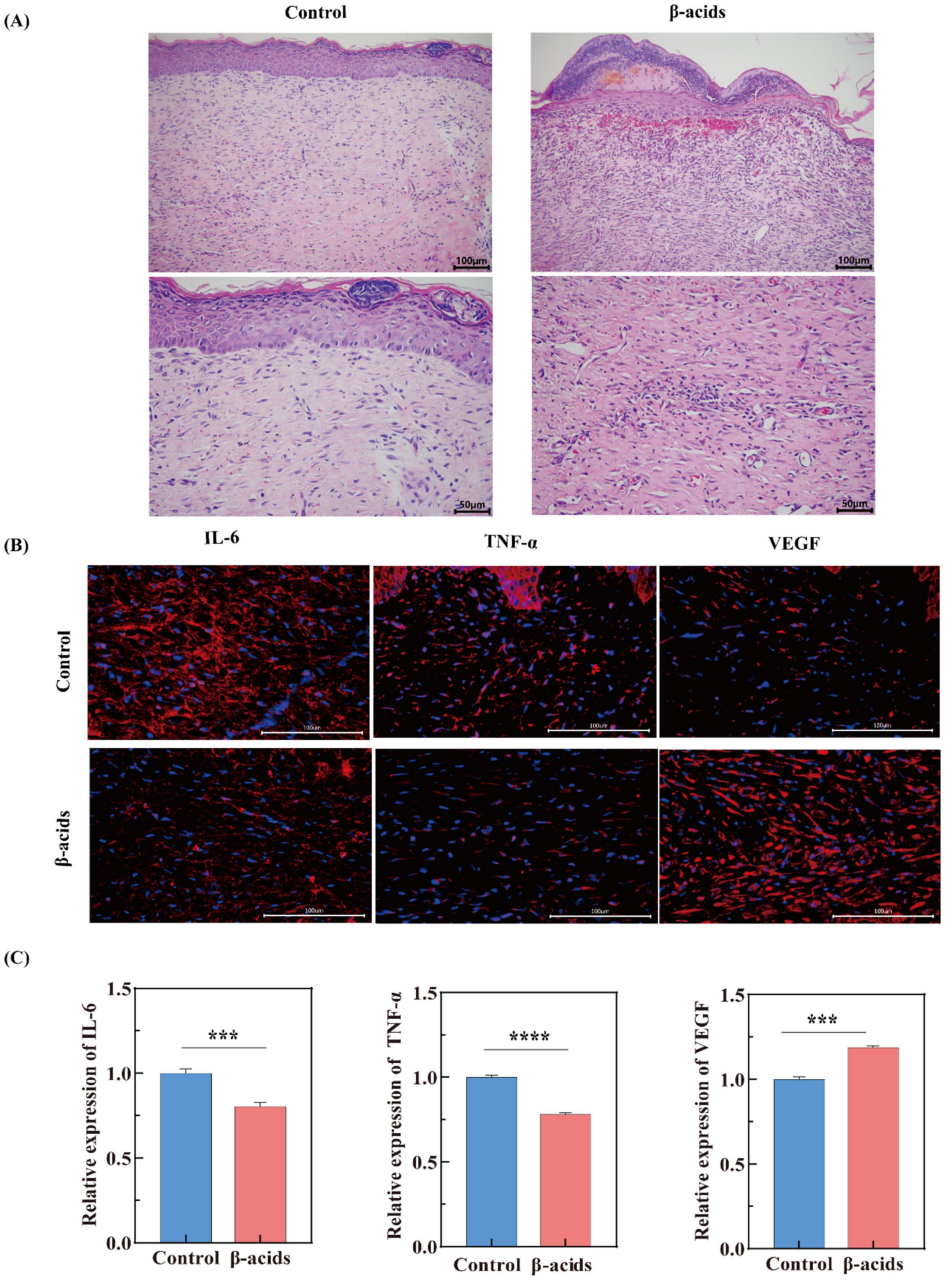
Evaluation of wound healing performance in Control and β-acids-treated groups via tissue section analysis. **(A)** H&E staining of wound tissues (scale bar: 100 μm and 50 μm). **(B)** Immunofluorescence staining of regenerated skin tissues after 15-day treatment with different samples, showing IL-6 (red), TNF-α (red), VEGF (red), and cell nuclei (blue) (scale bar: 100 μm). **(C)** Quantitative analysis of relative fluorescence intensity for IL-6, TNF-α, and VEGF in regenerated skin tissues. Data are presented as mean ± standard deviation from triplicate assays, with significant differences indicated by different letters (*p* < 0.05).

H&E staining was further used to evaluate the quality of regenerated skin tissue after 15 days of different treatments. As shown in Figure 8A, the epidermal layer over the wound in the control group was thinner than β-acids groups, with apparent defects in both epidermal and dermal structures and varying levels of inflammatory cell infiltration. This indicated underdeveloped dermal tissue, with loosely arranged and disorganized collagen fibers. In contrast, the treatment group exhibited an intact epithelial layer, regenerated dermal tissue containing hair follicles, and wounds that gradually assumed a mountain-like shape—likely due to contraction of newly formed peripheral tissue toward the wound center (Wang et al., 2022). Consistent with the wound area healing results, these findings further confirm the superior antimicrobial activity of β-acids and its potential to accelerate *in vivo* healing of infected wounds.

IL-6 is a pleiotropic cytokine critical in coordinating the inflammatory phase of wound healing (Das, 2023). IL-6 also stimulates keratinocyte proliferation and fibroblast function, bridging inflammation to tissue regeneration (Ditzen et al., 2023). However, persistent IL-6 elevation can exacerbate inflammation, impairing healing in chronic wounds. TNF-α drives early pro-inflammatory responses by enhancing vascular permeability, leukocyte adhesion, and phagocytosis (Wang et al., 2020). Additionally, VEGF is the master regulator of angiogenesis. It induces endothelial cell proliferation, migration, and new blood vessel formation, ensuring oxygen and nutrient delivery to the wound bed (Liu et al., 2024). VEGF also supports granulation tissue development and collagen organization. In infected wounds, adequate VEGF expression correlates with improved perfusion and resolution of inflammation (Shams et al., 2022). Subsequently, the expression levels of IL-6, TNF-α, and VEGF were evaluated using immunofluorescence staining techniques. After a period of 15 days, tissue samples from mouse trauma models were collected, processed, and stained for analysis. As depicted in Figure 8B, there was a marked reduction in the red fluorescence intensity of IL-6 and TNF-α in the treated group compared to the control group, suggesting a significant downregulation of IL-6 and TNF-α expression (Figure 8C).

It may be due to the release of pathogen-associated molecular patterns (PAMPs) after MRSA infection, which activate toll-like receptors (TLRs) on immune cells, triggering the NF-κB and MAPK pathways (Wang et al., 2023). This sustains high-level production of pro-inflammatory cytokines like IL-6 and TNF-α. However, when β-acids with antimicrobial effects was added, it reduced inflammatory tissue damage by inhibiting IL-6/TNF-α levels, thereby rebalancing the cytokine environment to accelerate wound repair. Besides, VEGF levels were significantly higher in the β-acids group compared with the control group, owing to the fact that upregulated VEGF promotes angiogenesis and regenerative processes (Beheshtizadeh et al., 2023). This shift reflects successful transition from pathogen clearance to tissue reconstruction. This result was also consistent with the H&E staining results. Overall, the results showed that β-acids exhibits significant antibacterial and anti-inflammatory effects, has good wound healing ability, and possesses the ability to promote the healing of infected wounds by decreasing the level of inflammatory factors and promoting tissue regeneration and repair.

## 4 Conclusion

In current study, β-acids was reported to consist of three main compounds: colupulone, lupulone and adlupulone by UPLC-Q-TOF/MS analysis. β-acids exhibited strong antimicrobial activity and was able to disrupt MRSA cell wall and cell membrane. Primary inhibitory mechanism of β-acids mainly inhibits MRSA growth by interfering with membrane permeability, membrane integrity, ion transport, energy metabolism and ROS homeostasis, while destroying bacterial proteins and activities. Moreover, the *in vivo* study in mice has shown obvious effects of promoting wound healing. Natural products exhibit unique advantages in overcoming drug resistance due to their structural diversity and multi-targeting properties. This study provides insights for the development of novel natural product-based antimicrobial drugs for the treatment of MRSA infections. Collectively, β-acids function as “dual-action agents”: eradicating MRSA through membrane targeting and oxidative stress, while concurrently resolving inflammation and accelerating tissue repair. However, there are still some shortcomings in this study: insufficient wound healing indicators have been introduced; lack of positive drugs; no further separation of β-acids was performed; lack of experimental verification for the interactions mentioned in molecular docking. And our future work could benefit from a comparative computational study for the standard to elucidate the precise determinants of binding affinity and specificity. Meanwhile, in future research, it is necessary to introduce more animal models to study wound repair and accelerate its clinical translation. Overall, β-acids is a promising candidate for treating multidrug-resistant wound infections, warranting further exploration of their clinical translation.

## Data Availability

The data supporting this study’s findings are available from the corresponding author upon reasonable request. Bulk RNA-seq data of β-acids-treated MRSA were provided in Sequence Read Archive (No. PRJNA1365699, http://www.ncbi.nlm.nih.gov/bioproject/1365699).

## References

[ref1] Ahmad M. Aduru S. V. Smith R. P. Zhao Z. Lopatkin A. J. (2025). The role of bacterial metabolism in antimicrobial resistance. Nat. Rev. Microbiol. 23, 439–454. doi: 10.1038/s41579-025-01155-0, PMID: 39979446 PMC12173792

[ref2] Assoni L. Milani B. Carvalho M. R. Nepomuceno L. N. Waz N. T. Guerra M. E. S. . (2020). Resistance mechanisms to antimicrobial peptides in Gram-positive bacteria. Front. Microbiol. 11:593215. doi: 10.3389/fmicb.2020.593215, PMID: 33193264 PMC7609970

[ref3] Averill-Bates D. (2024). Reactive oxygen species and cell signaling. Review. Biochim. Biophys. Acta Mol. Cell. Res. 1871:119573. doi: 10.1016/j.bbamcr.2023.119573 37949302

[ref4] Baeza N. Mercade E. (2021). Relationship between membrane vesicles, extracellular ATP and biofilm formation in Antarctic Gram-negative bacteria. Microb. Ecol. 81, 645–656. doi: 10.1007/s00248-020-01614-6, PMID: 33025062 PMC7982384

[ref5] Beheshtizadeh N. Gharibshahian M. Bayati M. Maleki R. Strachan H. Doughty S. . (2023). Vascular endothelial growth factor (VEGF) delivery approaches in regenerative medicine. Biomed. Pharmacother. 166:115301. doi: 10.1016/j.biopha.2023.115301, PMID: 37562236

[ref6] Broz P. Pelegrín P. Shao F. (2020). The gasdermins, a protein family executing cell death and inflammation. Nat. Rev. Immunol. 20, 143–157. doi: 10.1038/s41577-019-0228-2, PMID: 31690840

[ref7] Das U. N. (2023). Infection, inflammation, and immunity in sepsis. Biomolecules 13:1332. doi: 10.3390/biom13091332, PMID: 37759732 PMC10526286

[ref8] David M. Z. Daum R. S. (2010). Community-associated methicillin-resistant *Staphylococcus aureus*: epidemiology and clinical consequences of an emerging epidemic. Clin. Microbiol. Rev. 23, 616–687. doi: 10.1128/CMR.00081-09, PMID: 20610826 PMC2901661

[ref9] Deutscher J. Francke C. Postma P. W. (2006). How phosphotransferase system-related protein phosphorylation regulates carbohydrate metabolism in bacteria. Microbiol. Mol. Biol. Rev. 70, 939–1031. doi: 10.1128/MMBR.00024-06, PMID: 17158705 PMC1698508

[ref10] Ditzen B. Aguilar-Raab C. Winter F. Hernández C. Schneider E. Bodenmann G. . (2023). Effects of intranasal oxytocin and positive couple interaction on immune factors in skin wounds. Brain Behav. Immun. 107, 90–97. doi: 10.1016/j.bbi.2022.08.011, PMID: 36058418

[ref11] Fedosova N. U. Habeck M. Nissen P. (2022). Structure and function of Na, K-ATPase—the sodium-potassium pump. Compr. Physiol. 12, 2659–2679. doi: 10.1002/j.2040-4603.2022.tb00195.x, PMID: 34964112

[ref12] Foster T. J. (2019). Can β-lactam antibiotics be resurrected to combat MRSA? Trends Microbiol. 27, 26–38. doi: 10.1016/j.tim.2018.06.005, PMID: 30031590

[ref13] Gao L. Huang M. Xiong Q. Liang Y. Mi L. Jiang Y. . (2024). Antibacterial mechanism, control efficiency, and nontarget toxicity evaluation of actinomycin X2 against *Xanthomonas citri* subsp. *citri*. J. Agric. Food Chem. 72, 4788–4800. doi: 10.1021/acs.jafc.3c08600, PMID: 38377546

[ref14] Gao H. Wang Q. Qi Q. He W. Li W. (2024). Component analysis using UPLC-Q-TOF/MS and quality evaluation using fingerprinting and chemometrics for hops. Food Chem. 457:140113. doi: 10.1016/j.foodchem.2024.140113, PMID: 38901344

[ref15] Goedseels M. Michiels C. W. (2023). Cell envelope modifications generating resistance to hop beta acids and collateral sensitivity to cationic antimicrobials in *Listeria monocytogenes*. Microorganisms 11:2024. doi: 10.3390/microorganisms11082024, PMID: 37630584 PMC10457916

[ref16] Hu W. Li C. Dai J. Cui H. Lin L. (2019). Antibacterial activity and mechanism of *Litsea cubeba* essential oil against methicillin-resistant *Staphylococcus aureus* (MRSA). Ind. Crop. Prod. 130, 34–41. doi: 10.1016/j.indcrop.2018.12.078

[ref17] Kang S. Kong F. Shi X. Han H. Li M. Guan B. . (2020). Antibacterial activity and mechanism of lactobionic acid against *Pseudomonas fluorescens* and methicillin-resistant *Staphylococcus aureus* and its application on whole milk. Food Control 108:106876. doi: 10.1016/j.foodcont.2019.106876

[ref18] Kawasuji H. Nagaoka K. Tsuji Y. Kimoto K. Takegoshi Y. Kaneda M. . (2023). Effectiveness and safety of linezolid versus vancomycin, teicoplanin, or daptomycin against methicillin-resistant *Staphylococcus aureus* bacteremia: a systematic review and meta-analysis. Antibiotics 12:697. doi: 10.3390/antibiotics12040697, PMID: 37107059 PMC10135165

[ref19] Kolenc Z. Langerholc T. Hostnik G. Ocvirk M. Štumpf S. Pintarič M. . (2022). Antimicrobial properties of different hop (*Humulus lupulus*) genotypes. Plants 12:120. doi: 10.3390/plants12010120, PMID: 36616249 PMC9824274

[ref20] Kong Y. Yan H. Hu J. Dang Y. Han Z. Tian B. . (2024). Antibacterial activity and mechanism of action of osthole against *Listeria monocytogenes*. J. Agric. Food Chem. 72, 10853–10861. doi: 10.1021/acs.jafc.3c07931, PMID: 38708871

[ref21] Kubo I. Nihei K. I. Tsujimoto K. (2003). Antibacterial action of anacardic acids against methicillin resistant *Staphylococcus aureus* (MRSA). J. Agric. Food Chem. 51, 7624–7628. doi: 10.1021/jf034674f, PMID: 14664518

[ref22] Kukurugya M. A. Rosset S. Titov D. V. (2024). The Warburg effect is the result of faster ATP production by glycolysis than respiration. Proc. Natl. Acad. Sci. U.S.A. 121:e2409509121. doi: 10.1073/pnas.2409509121, PMID: 39514306 PMC11573683

[ref23] Lakhundi S. Zhang K. (2018). Methicillin-resistant *Staphylococcus aureus*: molecular characterization, evolution, and epidemiology. Clin. Microbiol. Rev. 31, 10–1128. doi: 10.1128/CMR.00020-18, PMID: 30209034 PMC6148192

[ref24] Leaper D. Assadian O. Edmiston C. E. (2015). Approach to chronic wound infections. Br. J. Dermatol. 173, 351–358. doi: 10.1111/bjd.13677, PMID: 25772951

[ref25] Lin L. Mao X. Sun Y. Cui H. (2018). Antibacterial mechanism of artemisinin/beta-cyclodextrins against methicillin-resistant *Staphylococcus aureus* (MRSA). Microb. Pathog. 118, 66–73. doi: 10.1016/j.micpath.2018.03.014, PMID: 29530805

[ref26] Liu H. Li S. Hua S. Wang H. Ping R. Zhang F. . (2025). Chitosan/PVA film containing β-acids/HP-β-CD inclusion complex for MRSA-infected wound healing. Int. J. Biol. Macromol. 319:145601. doi: 10.1016/j.ijbiomac.2025.145601, PMID: 40581014

[ref27] Liu X. Zeng J. Huang K. Wang J. (2019). Structure of the mannose transporter of the bacterial phosphotransferase system. Cell Res. 29, 680–682. doi: 10.1038/s41422-019-0194-z, PMID: 31209249 PMC6796895

[ref28] Liu J. Zhang Y. Liu C. Jiang Y. Wang Z. Guo Z. . (2024). A single dose of VEGF-A circular RNA sustains *in situ* long-term expression of protein to accelerate diabetic wound healing. J. Control. Release 373, 319–335. doi: 10.1016/j.jconrel.2024.07.018, PMID: 38986911

[ref29] Lu L. Hu W. Tian Z. Yuan D. Yi G. Zhou Y. . (2019). Developing natural products as potential anti-biofilm agents. Chin. Med. 14:11. doi: 10.1186/s13020-019-0232-2, PMID: 30936939 PMC6425673

[ref30] O’Toole G. A. (2011). Microtiter dish biofilm formation assay. J. Vis. Exp. 47:2437. doi: 10.3791/2437 PMC3182663 21307833

[ref31] Oh I. Yang W. Y. Park J. Lee S. Mar W. Oh K. B. . (2011). *In vitro* Na^+^/K^+^-ATPase inhibitory activity and antimicrobial activity of sesquiterpenes isolated from *Thujopsis dolabrata*. Arch. Pharm. Res. 34, 2141–2147. doi: 10.1007/s12272-011-1218-5, PMID: 22210041

[ref32] Peacock S. J. Paterson G. K. (2015). Mechanisms of methicillin resistance in *Staphylococcus aureus*. Annu. Rev. Biochem. 84, 577–601. doi: 10.1146/annurev-biochem-060614-034516, PMID: 26034890

[ref33] Peng B. O. Su Y. B. Li H. Han Y. I. Guo C. Tian Y. M. . (2015). Exogenous alanine and/or glucose plus kanamycin kills antibiotic-resistant bacteria. Cell Metab. 21, 249–262. doi: 10.1016/j.cmet.2015.01.008, PMID: 25651179

[ref34] Pranantyo D. Yeo C. K. Wu Y. Fan C. Xu X. Yip Y. S. . (2024). Hydrogel dressings with intrinsic antibiofilm and antioxidative dual functionalities accelerate infected diabetic wound healing. Nat. Commun. 15:954. doi: 10.1038/s41467-024-44968-y, PMID: 38296937 PMC10830466

[ref35] Ravera S. Tancreda G. Vezzulli L. Schito A. M. Panfoli I. (2023). Cirsiliol and quercetin inhibit ATP synthesis and decrease the energy balance in methicillin-resistant *Staphylococcus aureus* (MRSA) and methicillin-resistant *Staphylococcus epidermidis* (MRSE) strains isolated from patients. Molecules 28:6183. doi: 10.3390/molecules28176183, PMID: 37687012 PMC10488605

[ref36] Ritter T. K. Wong C. H. (2001). Carbohydrate-based antibiotics: a new approach to tackling the problem of resistance. Angew. Chem. Int. Edit. 40, 3508–3533. doi: 10.1002/1521-3773(20011001)40:19<3508::AID-ANIE3508>3.0.CO;2-I, PMID: 11592183

[ref37] Rossini F. Virga G. Loreti P. Iacuzzi N. Ruggeri R. Provenzano M. E. (2021). Hops (*Humulus lupulus* L.) as a novel multipurpose crop for the Mediterranean region of Europe: challenges and opportunities of their cultivation. Agriculture 11:484. doi: 10.3390/agriculture11060484

[ref38] Roy R. Tiwari M. Donelli G. Tiwari V. (2018). Strategies for combating bacterial biofilms: a focus on anti-biofilm agents and their mechanisms of action. Virulence 9, 522–554. doi: 10.1080/21505594.2017.1313372, PMID: 28362216 PMC5955472

[ref39] Shams F. Moravvej H. Hosseinzadeh S. Mostafavi E. Bayat H. Kazemi B. . (2022). Overexpression of VEGF in dermal fibroblast cells accelerates the angiogenesis and wound healing function: *in vitro* and in vivo studies. Sci. Rep. 12:18529. doi: 10.1038/s41598-022-23304-8, PMID: 36323953 PMC9630276

[ref40] Silver L. L. (2006). Does the cell wall of bacteria remain a viable source of targets for novel antibiotics? Biochem. Pharmacol. 71, 996–1005. doi: 10.1016/j.bcp.2005.10.029, PMID: 16290173

[ref41] Smith J. R. LeBlanc A. R. Wuest W. M. (2025). From natural products to small molecules: recent advancements in anti-MRSA therapeutics. ACS Med. Chem. Lett. 16, 542–551. doi: 10.1021/acsmedchemlett.5c00061, PMID: 40236547 PMC11995227

[ref42] Soheili M. Alinaghipour A. Salami M. (2022). Good bacteria, oxidative stress and neurological disorders: possible therapeutical considerations. Life Sci. 301:120605. doi: 10.1016/j.lfs.2022.120605, PMID: 35508256

[ref43] Sommella E. Verna G. Liso M. Salviati E. Esposito T. Carbone D. . (2021). Hop-derived fraction rich in beta acids and prenylflavonoids regulates the inflammatory response in dendritic cells differently from quercetin: unveiling metabolic changes by mass spectrometry-based metabolomics. Food Funct. 12, 12800–12811. doi: 10.1039/D1FO02361F, PMID: 34859812

[ref44] Spari D. Beldi G. (2020). Extracellular ATP as an inter-kingdom signaling molecule: release mechanisms by bacteria and its implication on the host. Int. J. Mol. Sci. 21:5590. doi: 10.3390/ijms21155590, PMID: 32759857 PMC7432876

[ref45] Sun J. Xie Y. Chen Z. Fan Y. Liu Y. Gao Q. . (2023). Antimicrobial activity and mechanism of *Magnolia officinalis* root extract against methicillin-resistant *Staphylococcus aureus* based on mannose transporter. Ind. Crop. Prod. 201:116953. doi: 10.1016/j.indcrop.2023.116953

[ref46] Tao Y. Qiao Q. Ruan Y. Fang X. Wang X. Zhang Y. . (2025). SIM imaging of bacterial membrane dynamics and lipid peroxidation during photodynamic inactivation with a dual-functional activatable probe. Chem. Sci. 16, 7766–7772. doi: 10.1039/d5sc00858a, PMID: 40191130 PMC11966534

[ref47] Tao J. Yan S. Zhou C. Liu Q. Zhu H. Wen Z. (2021). Total flavonoids from *Potentilla kleiniana* Wight et Arn inhibits biofilm formation and virulence factors production in methicillin-resistant *Staphylococcus aureus* (MRSA). J. Ethnopharmacol. 279:114383. doi: 10.1016/j.jep.2021.114383, PMID: 34214645

[ref48] Tian B. Cheng J. Zhang T. Liu Y. Chen D. (2022). Multifunctional chitosan-based film loaded with hops β-acids: preparation, characterization, controlled release and antibacterial mechanism. Food Hydrocoll. 124:107337. doi: 10.1016/j.foodhyd.2021.107337

[ref49] Tian B. Li W. Wang J. Liu Y. (2021). Functional polysaccharide-based film prepared from chitosan and β-acids: structural, physicochemical, and bioactive properties. Int. J. Biol. Macromol. 181, 966–977. doi: 10.1016/j.ijbiomac.2021.04.100, PMID: 33887287

[ref50] Tian B. Qiao X. Guo S. Li A. Xu Y. Cao J. . (2024). Synthesis of β-acids loaded chitosan-sodium tripolyphosphate nanoparticle towards controlled release, antibacterial and anticancer activity. Int. J. Biol. Macromol. 257:128719. doi: 10.1016/j.ijbiomac.2023.128719, PMID: 38101686

[ref51] Tian B. Xu D. Cheng J. Liu Y. (2021). Chitosan-silica with hops β-acids added films as prospective food packaging materials: preparation, characterization, and properties. Carbohydr. Polym. 272:118457. doi: 10.1016/j.carbpol.2021.118457, PMID: 34420717

[ref52] Tikhomirova A. Rahman M. M. Kidd S. P. Ferrero R. L. Roujeinikova A. (2024). Cysteine and resistance to oxidative stress: implications for virulence and antibiotic resistance. Trends Microbiol. 32, 93–104. doi: 10.1016/j.tim.2023.06.010, PMID: 37479622

[ref53] Turner N. A. Sharma-Kuinkel B. K. Maskarinec S. A. Eichenberger E. M. Shah P. P. Carugati M. . (2019). Methicillin-resistant *Staphylococcus aureus*: an overview of basic and clinical research. Nat. Rev. Microbiol. 17, 203–218. doi: 10.1038/s41579-018-0147-4, PMID: 30737488 PMC6939889

[ref54] Wang S. Liu Y. Sun Q. Zeng B. Liu C. Gong L. . (2023). Triple cross-linked dynamic responsive hydrogel loaded with selenium nanoparticles for modulating the inflammatory microenvironment via PI3K/Akt/NF-κB and MAPK signaling pathways. Adv. Sci. 10:2303167. doi: 10.1002/advs.202303167, PMID: 37740428 PMC10625091

[ref55] Wang Y. Niu W. Qu X. Lei B. (2022). Bioactive anti-inflammatory thermocatalytic nanometal-polyphenol polypeptide scaffolds for MRSA-infection/tumor postsurgical tissue repair. ACS Appl. Mater. Interfaces 14, 4946–4958. doi: 10.1021/acsami.1c21082, PMID: 35073045

[ref56] Wang X. Zhang S. Dong M. Li Y. Zhou Q. Yang L. (2020). The proinflammatory cytokines IL-1β and TNF-α modulate corneal epithelial wound healing through p16Ink4a suppressing STAT3 activity. J. Cell. Physiol. 235, 10081–10093. doi: 10.1002/jcp.29823, PMID: 32474927

[ref57] Weng Z. Zeng F. Wang M. Guo S. Tang Z. Itagaki K. . (2024). Antimicrobial activities of lavandulylated flavonoids in *Sophora flavences* against methicillin-resistant *Staphylococcus aureus* via membrane disruption. J. Adv. Res. 57, 197–212. doi: 10.1016/j.jare.2023.04.017, PMID: 37137428 PMC10918359

[ref58] Xu T. Tao X. He H. Kempher M. L. Zhang S. Liu X. . (2023). Functional and structural diversification of incomplete phosphotransferase system in cellulose-degrading clostridia. ISME J. 17, 823–835. doi: 10.1038/s41396-023-01392-2, PMID: 36899058 PMC10203250

[ref59] Yang X. Lan W. Xie J. (2022). Antimicrobial and anti-biofilm activities of chlorogenic acid grafted chitosan against *Staphylococcus aureus*. Microb. Pathog. 173:105748. doi: 10.1016/j.micpath.2022.105748, PMID: 36064104

[ref60] Yi L. Cao M. Chen X. Bai Y. Wang W. Wei X. . (2024). *In vitro* antimicrobial synergistic activity and the mechanism of the combination of naringenin and amikacin against antibiotic-resistant *Escherichia coli*. Microorganisms 12:1871. doi: 10.3390/microorganisms12091871, PMID: 39338545 PMC11433787

[ref61] Yuan Z. Ouyang P. Gu K. Rehman T. Zhang T. Yin Z. . (2019). The antibacterial mechanism of oridonin against methicillin-resistant *Staphylococcus aureus* (MRSA). Pharm. Biol. 57, 710–716. doi: 10.1080/13880209.2019.1674342, PMID: 31622118 PMC8871620

[ref62] Zeng J. Hong Y. Zhao N. Liu Q. Zhu W. Xiao L. . (2022). A broadly applicable, stress-mediated bacterial death pathway regulated by the phosphotransferase system (PTS) and the cAMP-Crp cascade. Proc. Natl. Acad. Sci. U.S.A. 119:e2118566119. doi: 10.1073/pnas.2118566119, PMID: 35648826 PMC9191683

[ref63] Zeng F. Shao S. Zou Z. Guo S. Cai Y. Yan C. . (2024). Multi-omics revealed antibacterial mechanisms of licochalcone A against MRSA and its antimicrobic potential on pork meat. Food Chem. X 24:101893. doi: 10.1016/j.fochx.2024.101893, PMID: 39498259 PMC11532437

[ref64] Zhang G. Zhang N. Yang A. Huang J. Ren X. Xian M. . (2021). Hop bitter acids: resources, biosynthesis, and applications. Appl. Microbiol. Biotechnol. 105, 4343–4356. doi: 10.1007/s00253-021-11329-4, PMID: 34021813

