# Supplementary material for: Antibacterial mechanism of hops β-acids against methicillin-resistant Staphylococcus aureus and promote wound healing
Source: Front Microbiol. 2025 Dec 3;16:1710545. doi: 10.3389/fmicb.2025.1710545 (PMC12708895; doi:10.3389/fmicb.2025.1710545)
Supplement: Supplementary file 1 [file Data_Sheet_1.docx]

**Antibacterial mechanism of hops** **β-acids against methicillin-resistant *Staphylococcus aureus*** **and promote wound healing**

Shuanghe Li ^a,b,1^, Shijie Wei ^b,1^, Feiyan Zhang ^a^, Qi Luo ^b^, Nan Yang ^b^, Xiao Zhang ^c^, Jiayue Liu ^d,*^, Xia Qiao ^a,*^, Bingren Tian ^a,*^

^a^ Ningxia Key Laboratory of Clinical and Pathogenic Microbiology, Surgery of Laboratory, Institute of Medical Sciences, General Hospital of Ningxia Medical University, Yinchuan, 750004, Ningxia, China

^b^ Pharmacy Department, General Hospital of Ningxia Medical University, Yinchuan, 750004, Ningxia, China

^c^ Sanitation Test Center, Pingliang Center for Disease Control and Prevention, Pingliang, 744000, Gansu, China

^d^ The State Key Laboratory of Mechanism and Quality of Chinese Medicine, Institute of Chinese Medical Sciences, University of Macau, Macao SAR, China

^1^ These authors contributed equally to this work and should be considered co-first authors.

***Corresponding authors:**

**Dr. Bingren Tian**

Email: tianbingren1@163.com

**Dr. Jiayue Liu**

Email: 15809513327@163.com

**Dr. Xia Qiao**

Email: xiaqiao1@sina.cn


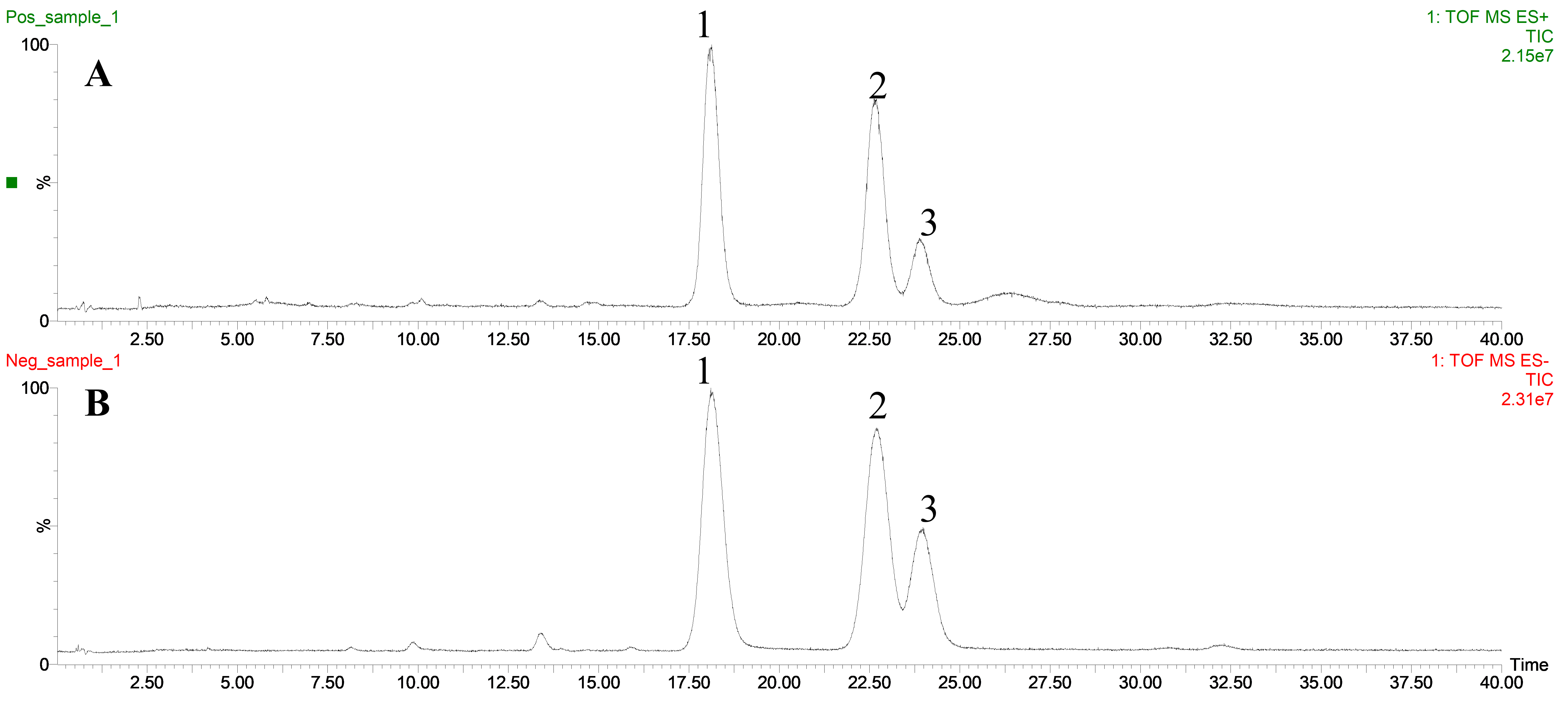


**Figure S1.** TIC of Hops β-acids in positive (A) and negative (B) ion mode. 1: colupulone; 2: lupulone; 3: adlupulone.


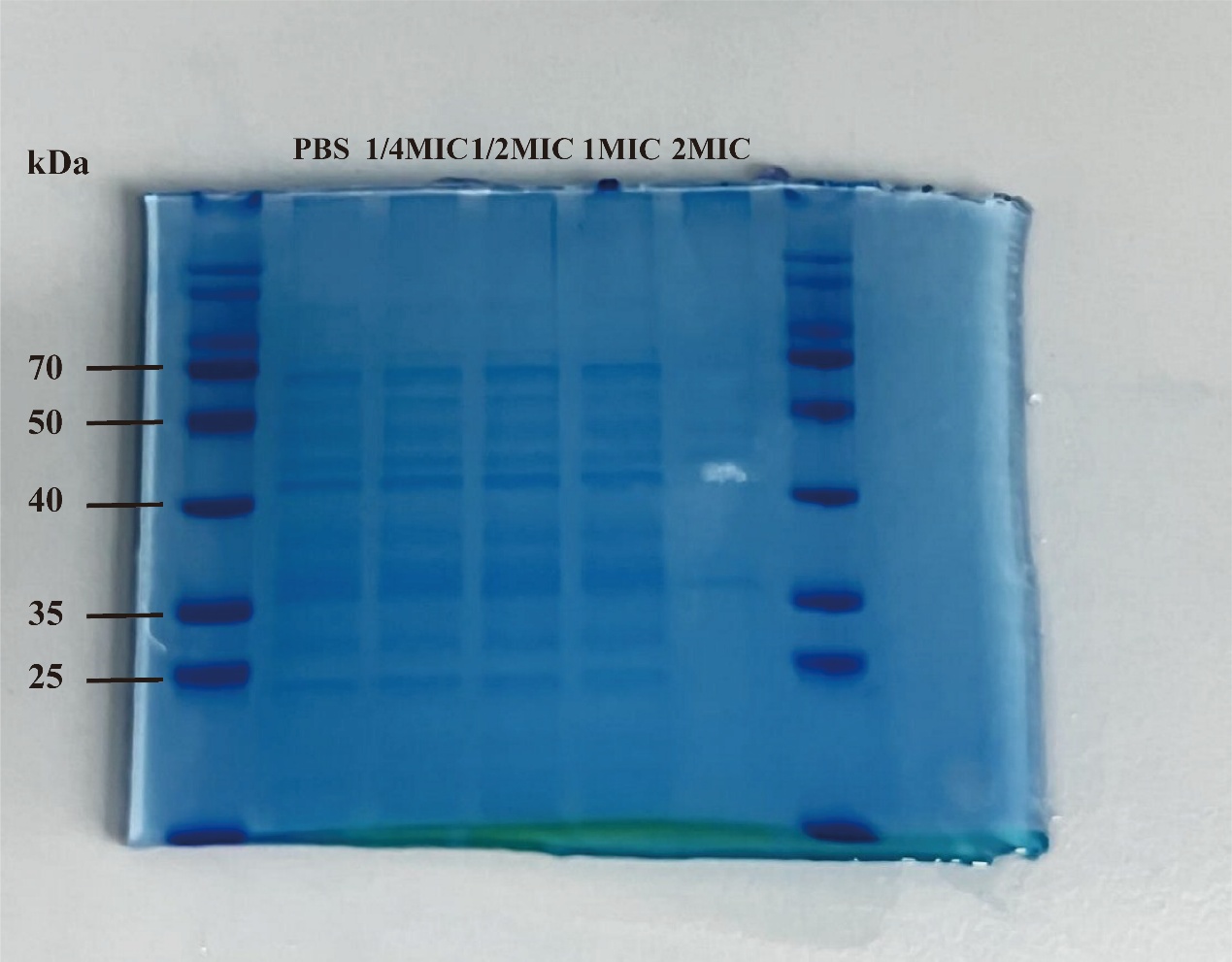


**Figure S2.** Raw data SDS-PAGE profile of proteins.


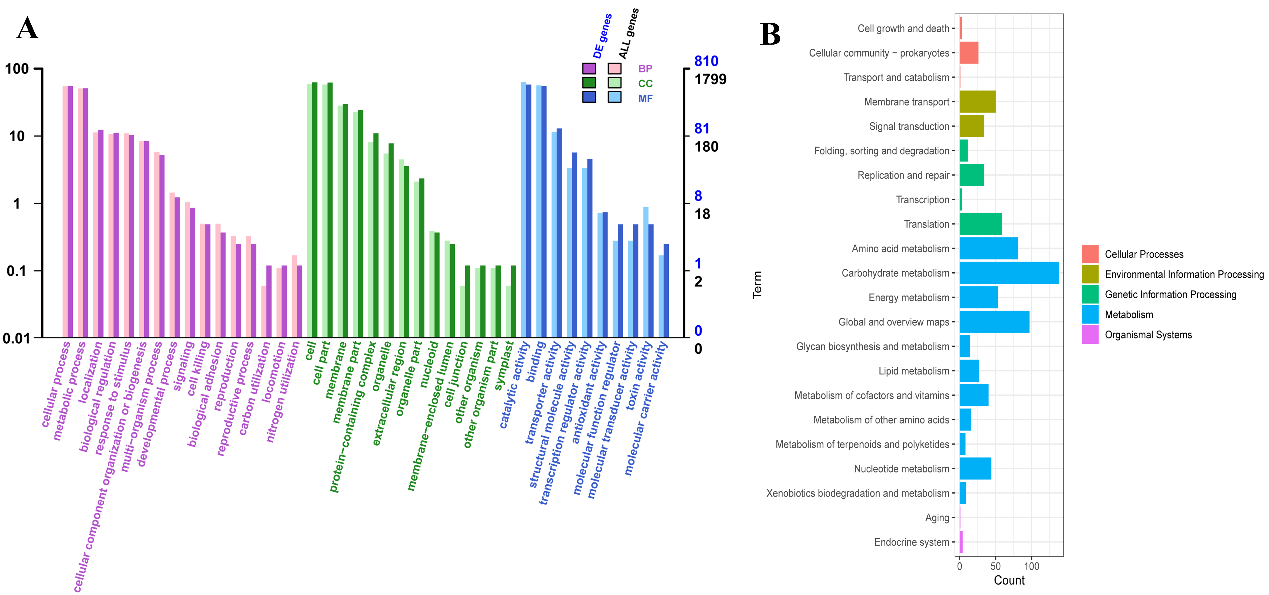


**Figure S3**. Differentially expressed genes of T-group and C-group in (A) GO terms and (B) KEGG pathways.

**Table S1.** The decreased expression of the main proteins of MRSA in the carbohydrate metabolism, amino acid metabolism, energy metabolism" and translation after the treatment of β-acids.

| Pathway | Gene ID | Protein ID | Protein symbol | Protein name | Fold change | P-value |
| --- | --- | --- | --- | --- | --- | --- |
| Carbohydrate metabolism | gene-SAR_RS02885 | WP_000726734.1 | araB | L-ribulokinase | 1.1509 | 0.00829 |
|  | gene-SAR_RS06520 | WP_000971307.1 | GK | glycerol kinase | 1.6703 | 3.89E-16 |
|  | gene-SAR_RS11925 | WP_000742891.1 | lacC | tagatose 6-phosphate kinase | 3.0055 | 3.03E-25 |
|  | gene-SAR_RS13560 | WP_0007726541.1 | idnK | gluconokinase | 1.8998 | 1.05E-20 |
| Amino acid metabolism | gene-SAR_RS03035 | 3ZFZ | BP2a | Penicillin-binding protein 2a | 1.7822 | 7.97E-20 |
|  | gene-SAR_RS06850 | NP_371548.1 | TC.AAT | amino acid transporter, AAT family | 4.8021 | 1.15E-163 |
|  | gene-SAR_RS07405 | NP_371189.1 | TC.APA | basic amino acid/polyamine antiporter, APA family | 2.9252 | 9.71E-32 |
|  | gene-SAR_RS09005 | WP_000744256.1 | lysP | lysine-specific permease | 1.5931 | 8.60E-11 |
| Energy metabolism | gene-SAR_RS08420 | WP_000722650.1 | era, ERAL1 | GTPase | 1.8940 | 2.50E-21 |
| Translation | gene-SAR_RS09000 | WP_001240899.1 | infC, MTIF3 | translation initiation factor IF-3 | 1.4563 | 7.36E-05 |
|  | gene-SAR_RS12095 | WP_00722635.1 | infA | translation initiation factor IF-1 | 1.9698 | 1.12E-20 |
